# Trends in migraine-specific medication consumption: a multinational analysis of 73 countries based on IQVIA-MIDAS database, 2015 to 2024

**DOI:** 10.1186/s10194-026-02363-6

**Published:** 2026-04-15

**Authors:** Cuiling Wei, Qiwen Fang, Yifang Huang, Wenxin Tian, Song Song, Xinya Mu, Rachel Yui Ki Chu, Yuqi Hu, Zijie Xu, Xue Li, Ian Chi Kei Wong, Esther Wai Yin Chan, Francisco Tsz Tsun Lai

**Affiliations:** 1 Department of Pharmacology and Pharmacy, Centre for Safe Medication Practice and Research, Li Ka Shing Faculty of Medicine, The University of Hong Kong, Room 113, 1/F, Estate Building 10 Sassoon Road, Pok Fu Lam, Hong Kong SAR China; 2 Department of Medicine, School of Clinical Medicine, Li Ka Shing Faculty of Medicine, The University of Hong Kong, Pok Fu Lam, Hong Kong SAR China; 3 Aston School of Pharmacy, Aston University, Birmingham, UK; 4 The University of Hong Kong Shenzhen Institute of Research and Innovation, Shenzhen, China; 5 Department of Pharmacy, The University of Hong Kong-Shenzhen Hospital, Shenzhen, China; 6 Department of Family Medicine and Primary Care, School of Clinical Medicine, Li Ka Shing Faculty of Medicine, The University of Hong Kong, Pok Fu Lam, Hong Kong SAR China; 7 Advanced Data Analytics for Medical Science (ADAMS) Limited, Hong Kong SAR, China; 8 Department of Pharmacology and Pharmacy Li Ka Shing Faculty of Medicine, The University of Hong Kong, 2/F, Laboratory Block, 21 Sassoon Road, Pok Fu Lam, Hong Kong SAR China

## Abstract

**Background:**

Migraine is a highly prevalent neurological disorder and one of the leading causes of disability worldwide. Despite this immense public health impact, real-world utilization of migraine-specific pharmacotherapies across different income levels remains poorly characterized. This study examines longitudinal consumption trends of migraine-specific medications (MSMs) across 73 countries.

**Methods:**

Using the IQVIA-MIDAS database, region/country-level quarterly sales volumes of specific MSMs and propranolol from 2015 to 2024 were extracted and normalized to standard units per 1000 population. Descriptive statistics were summarized using the median, interquartile range (IQR), population-weighted mean and standard error (SE). For stratified analysis, the 73 countries were classified into high-, upper-middle-, and lower-middle-income countries (HICs, UMICs, LMICs). Temporal trends were evaluated using joinpoint regression to calculate the annual percent change (APC). Multiple intervention interrupted time series (ITS) models assessed the sequential impacts of CGRP introduction (May 2018) and the COVID-19 pandemic (January 2020).

**Results:**

Between 2015 and 2024, median consumption of all MSMs nearly doubled from 101.77 to 194.39, with population-weighted averages rising from 149.23 to 201.05. The MSM consumption was overwhelmingly concentrated in HICs (337.11 to 378.13), while consumption in UMICs (9.40 to 15.10) and LMICs (18.77 to 28.01) remained persistently low. Acute MSMs, mainly triptans, drove most consumption and showed accelerating growth. Preventive MSM consumption initially declined before rebounding, largely from rapid uptake of CGRP inhibitors in HICs. Propranolol remained the most consistently consumed preventive agent across all income groups. The COVID-19 pandemic coincided with a temporary drop in MSM consumption followed by accelerated long-term growth in several groups.

**Conclusions:**

MSM consumption remains markedly unequal and heavily concentrated in HICs. The advanced CGRP inhibitors represent an important therapeutic advance, while underlying gaps in migraine-specific preventive pharmacotherapy largely unresolved. Addressing these global disparities requires healthcare prioritization aligned with the actual disability weight of migraine.

**Supplementary Information:**

The online version contains supplementary material available at 10.1186/s10194-026-02363-6.

## Introduction

Migraine is a complex neurological disorder clinically distinguished from other primary headaches by recurrent attacks of moderate-to-severe, unilateral, pulsating pain, which are characteristically accompanied by nausea, sensory hypersensitivities (such as photophobia and phonophobia), and exacerbation during routine physical activity [1]. The onset and severity of these debilitating attacks are frequently precipitated or exacerbated by a complex interplay of endogenous and exogenous triggers, most notably psychosocial stress, hormonal fluctuations, sleep disturbances, and specific environmental or dietary factors [2]. Although current evidence indicates that migraine does not significantly increase all-cause mortality [3] subtypes like migraine with aura are independent risk factors for ischemic stroke and cardiovascular events [4, 5]. Migraine remains one of the most prevalent neurological disorders and a leading contributor to disability adjusted life years worldwide, disproportionately affecting women and working-age adults, with substantial impacts on daily functioning, work productivity, direct healthcare costs, and indirect societal costs [6–9]. The Global Burden of Diseases, Injuries, and Risk Factors Study (GBD) 2023 estimates indicate a global migraine prevalence of approximately 14% with 487.5 years lived with disability (YLDs) per 100,000 population in 2023 [10]. Notably, the study also showed that migraine-attributed disability remains relatively consistent across different regions and has shown no substantial variation over time [10].

Major international and national guidelines are largely aligned in their recommendations for migraine pharmacotherapy. For acute treatment, nonsteroidal anti-inflammatory drugs (NSAIDs, e.g., ibuprofen) or acetaminophen/paracetamol are recommended for mild to moderate attacks, and triptans (e.g., sumatriptan) for moderate to severe attacks, with anti-emetics as adjuncts and strict avoidance of opioids [11–13]. For prevention, patients meeting guideline established thresholds for attack frequency or disability are recommended to receive beta-blockers such as propranolol, antiepileptics such as topiramate and valproate, or amitriptyline as first-line options [12, 14]. Calcitonin gene-related peptide (CGRP) inhibitors, which are more recently developed and marketed, are currently positioned as first-line treatments in high-resource settings [15], whereas in many other countries initiation of CGRP inhibitors is restricted to patients who have used at least two conventional preventive medicines without satisfactory response [16, 17]. However, real-world utilization studies suggest a clear gap between guidelines and actual practice. Acute pharmacological treatment is largely accounted for NSAIDs [18, 19], while studies from Europe and Asia suggest that a substantial proportion of patients with moderate to severe migraine do not receive triptans, despite guideline recommendations [19, 20]. Preventive pharmacotherapy remains underused, with less than 30% of eligible patients treated with poor adherence [21–23]. Although CGRP inhibitors demonstrate substantial reductions in migraine days and decrease the need for acute medication in trials and real-world studies [24, 25], their clinical penetration in sampled populations remains less than 7% of patients formally eligible for prophylactic treatment [18, 26].

Existing evidence on antimigraine pharmacotherapy remains fragmented, mostly limited to single-country or regional claims data with short follow-up periods, and rarely provides standardized multinational comparisons of longitudinal consumption and cost trends to identify unmet needs and to assess inequity to medication access. Furthermore, little is known about how the introduction of CGRP inhibitors after 2018 has reshaped MSM utilization across diverse healthcare systems. To address this gap, we used the IQVIA Multinational Integrated Data Analysis System (MIDAS) database to examine trends in the consumption of migraine-specific medications (MSMs) across 73 countries from 2015 to 2024, spanning the pre- and post-CGRP periods. We aim to characterize the temporal and geographic patterns and to assess the macro-level shifts associated with CGRP introduction on MSM consumption. Understanding these multinational MSM consumption patterns provides critical empirical evidence across key stakeholders within the medical community. For professional societies and guideline committees, characterizing real-world utilization shifts is essential to identify potential gaps in guideline implementation and systemic under-treatment, thereby guiding targeted medical education and advocacy efforts. Similarly, these findings enable policymakers and payers to assess global disparities in MSM access and ultimately optimize resource allocation.

## Methods

### Data source

The IQVIA-MIDAS serves as a comprehensive platform for tracking pharmaceutical market dynamics, aggregating audited sales data from a wide array of global suppliers, including wholesalers, retail outlets, and hospital networks. This database captures detailed metrics on drug volumes across diverse healthcare settings, enabling reliable cross-country comparisons through standardized methodologies. Accordingly, consumption within the context of our study is operationally defined by these aggregated sales volumes, representing national-level drug utilization. For this study, we utilized quarterly sales data in 73 countries/regions from IQVIA-MIDAS spanning January 2015 to December 2024. To adjust for demographic variations, country-level population estimates were extracted from the World Bank World Development Indicators for each corresponding year [27]. As detailed in Table S1, countries included in this study were stratified into high-income countries/regions (HICs, *N* = 41), upper-middle-income countries/regions (UMICs, *N* = 20), and lower-middle-income countries/regions (LMICs, *N* = 12) based on the World Bank 2024’s classification by national income level [28].

### Definition and selection of MSMs

MSMs in this analysis were defined using the Anatomical Therapeutic Chemical (ATC) classification code N02C, which encompasses specific antimigraine preparations such as ergot alkaloids, triptans, and CGRP inhibitors. We additionally included propranolol, a beta-blocker classified under the ATC code C07A, as it is the only preventive agent explicitly listed for migraine in the World Health Organization Essential Medicines List (WHO EML) [29] and is widely recommended as a first-line preventive medicine in guidelines [14]. This focused scope excludes non-specific acute treatments like NSAIDs or acetaminophen, and other repurposed preventives such as topiramate, valproate, or amitriptyline, because they are primarily indicated for other conditions and their inclusion would inflate estimates by capturing off-label or non-migraine-related use. Methysergide was excluded from the analysis because it has been broadly discontinued from the global market due to safety concerns during the study period [30]. The list of MSMs included in our study was provided in Table S2.

### Data analysis

To facilitate valid cross-country comparisons, quarterly consumption volumes were aggregated into annual totals and subsequently normalized to standard units per 1000 population, yielding standardized country-level consumption. Based on these standardized metrics, descriptive statistics were conducted to summarize the data distributions with consumption volumes expressed as medians and interquartile ranges (IQRs) to account for potential skewness in cross-national data. For overall and income-level group analyses, group-level consumption was primarily determined using the population-weighted average to account for substantial variations in population sizes across countries. Specifically, each country’s consumption was weighted by its corresponding population size for the respective year. To provide a comprehensive descriptive overview, the simple unweighted average consumption across countries within each category was also calculated. To quantify the variability of these estimates, both weighted and unweighted means were reported alongside their respective standard errors (SEs). The SE for the population-weighted mean was derived by calculating the square root of the unbiased population-weighted sample variance, divided by the square root of the number of contributing countries in that category.

To evaluate the long-term temporal trends, joinpoint regression analysis was performed using the population-weighted average consumption to identify significant inflection points where the trend of drug consumption shifts [31]. Specifically, the natural logarithm of consumption was modeled as a continuous function of time. We evaluated candidate models allowing for varying numbers of joinpoints and selected the optimal model yielding the minimum Bayesian Information Criterion (BIC) to avoid over-parameterization. Since our study period covered only ten years, the number of joinpoints was limited to a maximum of two. To prevent overfitting, we enforced a minimum interval of three years between consecutive joinpoints or study period boundaries. For each identified segment, the annual percent change (APC) and its corresponding 95% confidence interval (CI) were calculated using the formula $\:APC=\left[\mathrm{exp}\left(\beta\:\right)-1\right]\times\:100$, where $\:\beta\:$ denotes the estimated slope of the regression line, thereby providing a standardized measure of temporal trend variations.

### Exploratory analysis

To assess the impact of two major macro-level events, we applied a three-phase single-arm interrupted time series (ITS) analysis using segmented regression models on the population-weighted average consumption [32]. To increase the number of data points and capture temporal variations more precisely, the analysis was conducted using quarterly consumption data. The two events evaluated were the advent of CGRP inhibitors and the onset of the COVID-19 pandemic (January 2020). For the CGRP introduction, May 2018 was explicitly utilized as a global proxy intervention point, as it marks the first regulatory approval of a CGRP-targeted therapy (erenumab by the United States Food and Drug Administration) [33].While actual regulatory approval, reimbursement, and clinical uptake timelines varied substantially across different countries, this timepoint serves as a uniform reference point. Methodologically, selecting an earlier date was precluded by the absence of any approved agents. Conversely, selecting a later, country-specific date (e.g., mid-to-late 2019) would severely truncate the intermediate phase prior to the COVID-19 pandemic, leaving an insufficient number of quarterly data points to reliably model any robust trend shift. Given the sequential nature of these events, we constructed a multiple intervention segmented regression model. Accordingly, the study timeframe was structured to incorporate a pre-intervention baseline period (2015 to 2018 Q1), an intermediate phase following the CGRP introduction but preceding the pandemic (2018 Q2 to 2019 Q4), and a post-COVID-19 onset period (2020 Q1 to 2024 Q4). The ITS models were configured to estimate both the immediate changes (level changes) and gradual trajectory changes (slope changes) in consumption sequentially following each respective event. Furthermore, the models were explicitly adjusted for underlying seasonality and potential autocorrelation to ensure the robustness of the estimates, as detailed in Supplementary Methods.

All analyses were performed using R version 4.2.0. Two-sided P-values < 0.05 were considered statistically significant.

## Results

### Overall MSM consumption trend

**Fig. 1:**
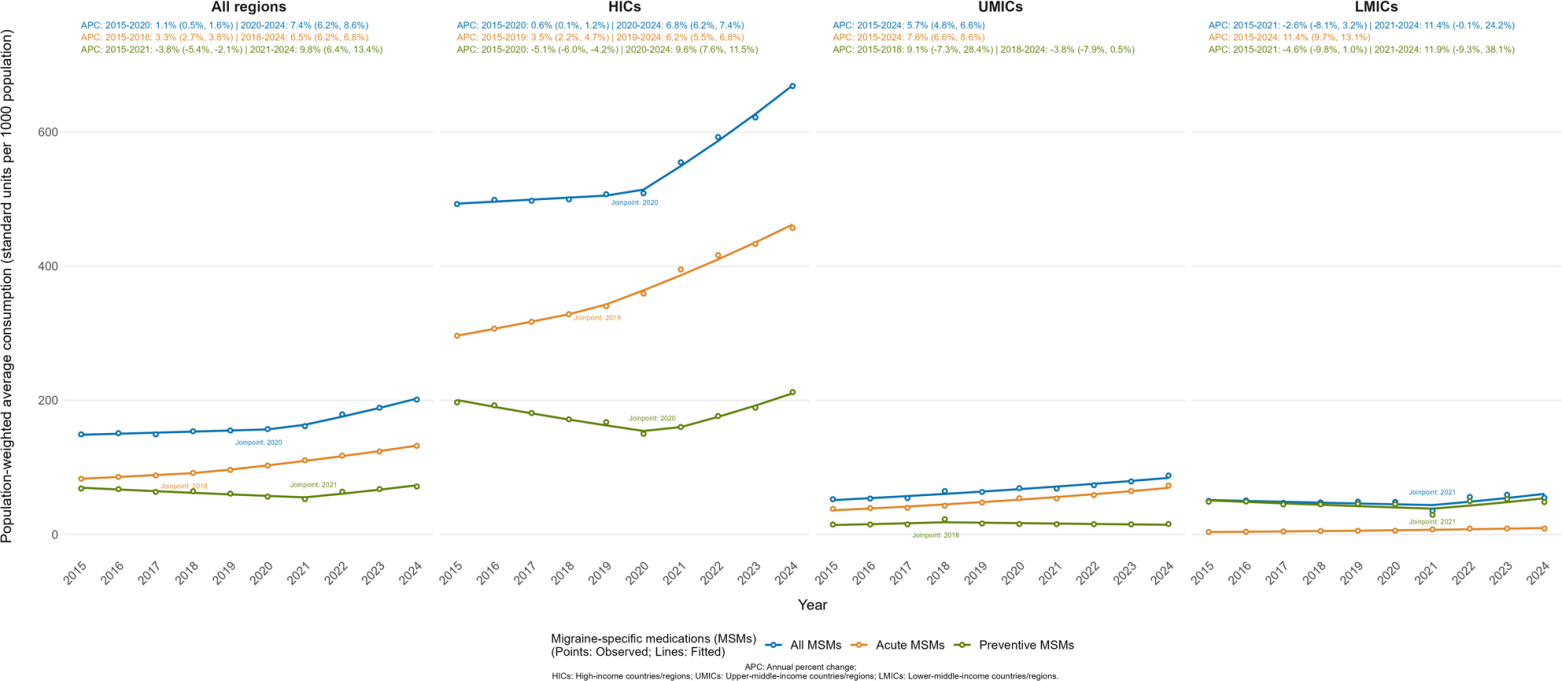
Trends in population-weighted average consumption of migraine-specific medications by income-level group, 2015–2024

Across the 73 countries/regions included in this study, the median consumption of all MSMs almost doubled from 101.77 (IQR: 12.40-444.31) standard units per 1,000 population in 2015 to 194.39 (IQR: 29.56-418.62) in 2024 (Table 1). The population-weighted average consumption rose from 149.23 (SE: 37.80) to 201.05 (SE: 44.09) over the decade. As shown in Fig. 1, this overall trend was characterized by modest initial growth (APC: 1.1%, 95% CI: 0.5% to 1.6%) from 2015 to 2020, followed by an accelerated upward trend through 2024 (APC: 7.4%, 95% CI: 6.2% to 8.6%). Detailed annual trends in both unweighted average and median consumption of overall MSMs stratified by income-level groups are further illustrated in Fig. S1a-b. In HICs, the population-weighted mean increased from 492.62 (SE: 76.43) to 668.61 (SE: 72.11) mirroring the overall trend (2015–2020 APC: 0.6%, 95% CI: 0.1% to 1.2%; 2020–2024 APC: 3.4%, 95% CI: 0.8% to 6.1%). The United Kingdom (2,016.41 in 2015; 1,859.33 in 2024) consistently recorded the highest consumption rates, whereas Uruguay (4.56 in 2015; 12.34 in 2024) consistently recorded the lowest levels throughout the study period. In MICs, consumption remained substantially lower but trended upward. UMICs exhibited a consistent growth (APC: 4.5%, 95% CI: 2.9% to 6.1%), with the population-weighted mean rising from 52.43 (SE: 33.97) to 87.84 (SE: 58.72). Brazil emerged as the top consumer by the end of the decade, exhibiting sustained growth from 406.84 in 2015 to 764.49 in 2024. Other highly populated countries in this group maintained lower consumption but exhibited divergent trends, with China experiencing a gradual increase (0.75 in 2015; 5.64 in 2024) and Indonesia a decline (8.94 in 2015; 0.39 in 2024). Similarly, LMICs showed a relatively flat overall trend (2015–2021 APC: -2.6%, 95% CI: -8.1% to 3.2%; 2021–2024 APC: 11.4%, 95% CI: -0.1% to 24.2%), changing from the population-weighted mean of 50.04 (SE: 40.49) to 54.70 (SE: 39.62). Tunisia was an outlier in this group with exceptionally high, albeit declining consumption (932.47 in 2015; 705.48 in 2024), while Vietnam (0.22 in 2015; 0.10 in 2024) steadily recorded the lowest consumption across the decade. Detailed country-level data on consumption of all MSMs from 2015 to 2024 were provided in Table S3.

**Table 1:** Consumption of migraine-specific medications and propranolol by income-level group, 2015–2024

Metrics	2015	2016	2017	2018	2019	2020	2021	2022	2023	2024
**a) All migraine-specific medications**										
**All regions**										
Number of countries	73	73	73	73	73	73	73	73	73	73
Median (IQR^a^)	101.77 (12.40–444.31)	115.30 (14.32–419.83)	120.30 (11.61–433.06)	123.65 (12.11–451.79)	119.47 (14.93–414.76)	127.84 (16.83–375.78)	137.00 (24.54–425.35)	159.14 (25.29–433.59)	188.33 (29.73–428.44)	194.39 (29.56–418.62)
Unweighted mean (SE^b^)	301.21 (50.06)	309.51 (56.44)	309.63 (57.01)	295.13 (45.68)	281.80 (43.93)	284.16 (42.63)	291.65 (43.64)	301.06 (43.65)	308.17 (43.30)	323.69 (45.48)
Population-weighted mean (SE)^c^	149.23 (37.80)	151.00 (38.39)	149.26 (37.61)	153.88 (36.90)	155.00 (36.98)	157.12 (35.84)	161.42 (37.74)	179.00 (39.78)	188.89 (41.20)	201.05 (44.09)
**High-income countries/regions**										
Number of countries	41	41	41	41	41	41	41	41	41	41
Median (IQR)	337.11 (101.77–585.69)	257.05 (105.09–607.93)	279.82 (120.30–577.57)	283.25 (123.65–687.36)	298.00 (119.47–544.58)	301.87 (127.84–569.79)	292.33 (146.49–577.25)	327.97 (159.14–589.86)	329.32 (188.33–595.24)	378.13 (194.39–639.79)
Unweighted mean (SE)	464.68 (76.29)	480.04 (89.13)	482.26 (90.44)	444.69 (67.37)	426.32 (65.44)	423.61 (61.59)	443.69 (63.88)	455.22 (63.65)	464.13 (62.31)	490.65 (65.72)
Population-weighted mean (SE)	492.62 (76.43)	498.92 (78.07)	497.62 (75.54)	499.63 (69.82)	507.32 (69.86)	508.72 (62.56)	554.93 (65.62)	592.58 (67.73)	621.78 (67.71)	668.61 (72.11)
**Upper-middle-income countries/regions**										
Number of countries	20	20	20	20	20	20	20	20	20	20
Median (IQR)	9.40 (2.36–50.07)	8.90 (3.01–44.98)	9.04 (3.86–53.35)	9.75 (4.06–68.45)	10.18 (3.27–65.66)	10.63 (5.16–69.64)	11.60 (4.83–79.71)	13.08 (4.40–89.72)	14.93 (4.13–105.16)	15.10 (5.93–119.27)
Unweighted mean (SE)	67.96 (29.22)	69.50 (30.69)	71.48 (31.32)	93.88 (44.93)	80.76 (35.04)	84.93 (36.91)	85.41 (35.26)	90.69 (37.55)	96.54 (40.04)	105.76 (43.46)
Population-weighted mean (SE)	52.43 (33.97)	53.50 (35.29)	54.18 (35.88)	64.64 (44.26)	63.31 (42.59)	68.98 (47.36)	68.37 (45.90)	73.42 (49.64)	79.06 (53.64)	87.84 (58.72)
**Lower-middle-income countries/regions**										
Number of countries	12	12	12	12	12	12	12	12	12	12
Median (IQR)	18.77 (6.47–124.16)	22.75 (8.16–141.19)	21.28 (6.83–131.87)	19.36 (5.09–95.29)	15.53 (5.34–96.80)	24.07 (4.86–108.92)	25.10 (7.69–82.61)	24.48 (7.43–115.48)	29.50 (7.10–112.15)	28.01 (8.37–112.53)
Unweighted mean (SE)	131.44 (76.45)	126.91 (68.17)	116.74 (61.06)	119.59 (70.73)	123.11 (72.00)	139.75 (85.81)	115.89 (71.42)	124.98 (67.67)	128.03 (67.25)	116.48 (59.08)
Population-weighted mean (SE)	50.04 (40.49)	50.68 (39.23)	47.06 (35.91)	47.74 (38.22)	48.87 (39.13)	48.49 (41.40)	35.31 (32.42)	55.82 (41.66)	59.05 (44.14)	54.70 (39.62)
**b) Acute migraine-specific medications**										
**All regions**										
Number of countries	73	73	73	73	73	73	73	73	73	73
Median (IQR^a^)	52.65 (5.90–240.27)	44.51 (7.06–246.05)	56.10 (7.14–252.21)	62.28 (7.12–256.69)	66.00 (8.28–256.06)	67.19 (10.22–270.41)	76.53 (10.85–290.13)	94.67 (11.48–324.62)	113.08 (15.33–328.30)	119.44 (16.48–359.61)
Unweighted mean (SE^b^)	151.79 (24.86)	156.92 (25.50)	163.09 (26.23)	169.97 (26.99)	178.61 (28.01)	187.40 (29.09)	198.22 (30.37)	206.73 (30.98)	218.39 (32.65)	229.93 (33.76)
Population-weighted mean (SE)^c^	83.12 (20.20)	85.79 (20.75)	88.05 (21.12)	91.77 (21.87)	96.21 (22.86)	102.68 (24.61)	110.55 (26.15)	117.51 (27.58)	123.64 (28.70)	132.07 (30.39)
**High-income countries/regions**										
Number of countries	41	41	41	41	41	41	41	41	41	41
Median (IQR)	181.47 (78.27–339.06)	185.13 (75.96–372.18)	196.57 (94.14–378.27)	207.38 (101.83–393.01)	235.31 (106.79–423.20)	250.29 (113.29–418.54)	264.26 (123.95–456.61)	281.87 (131.66–466.57)	296.60 (141.76–473.64)	312.72 (148.58–485.63)
Unweighted mean (SE)	252.94 (36.05)	261.13 (36.89)	271.33 (37.82)	281.57 (38.71)	294.42 (39.87)	306.10 (41.22)	323.63 (43.12)	336.29 (43.36)	353.38 (45.75)	369.28 (46.84)
Population-weighted mean (SE)	296.21 (31.07)	306.93 (31.47)	317.05 (31.42)	328.40 (31.70)	340.27 (31.83)	359.10 (33.83)	395.18 (36.63)	416.49 (37.72)	433.19 (37.50)	456.94 (38.14)
**Upper-middle-income countries/regions**										
Number of countries	20	20	20	20	20	20	20	20	20	20
Median (IQR)	7.52 (2.36–11.42)	6.90 (2.91–10.91)	6.83 (3.75–11.39)	6.96 (4.06–11.51)	7.54 (2.97–13.58)	8.31 (3.80–18.04)	9.29 (4.51–24.16)	11.33 (4.40–29.83)	13.87 (4.13–36.90)	13.86 (5.50–45.58)
Unweighted mean (SE)	32.13 (20.10)	33.05 (20.76)	34.29 (21.02)	37.45 (22.84)	41.69 (25.83)	48.10 (29.56)	50.25 (28.93)	55.62 (31.49)	62.26 (34.33)	71.41 (38.12)
Population-weighted mean (SE)	38.25 (30.44)	39.22 (31.40)	39.64 (31.75)	42.83 (34.49)	47.65 (38.96)	54.00 (44.52)	53.62 (43.40)	58.72 (47.25)	64.64 (51.45)	72.81 (56.71)
**Lower-middle-income countries/regions**										
Number of countries	12	12	12	12	12	12	12	12	12	12
Median (IQR)	3.13 (1.68–7.39)	3.58 (1.75–9.72)	6.21 (2.61–10.65)	5.95 (3.04–11.79)	10.04 (3.24–15.23)	11.14 (3.64–17.75)	14.64 (3.77–26.71)	9.99 (4.96–27.62)	12.91 (5.39–30.36)	16.46 (5.68–30.38)
Unweighted mean (SE)	5.66 (2.05)	7.32 (2.35)	7.91 (2.14)	9.55 (2.79)	11.12 (2.98)	14.02 (3.92)	16.33 (3.99)	15.93 (3.99)	17.38 (4.30)	18.00 (4.24)
Population-weighted mean (SE)	3.69 (0.90)	4.02 (1.16)	4.46 (1.26)	5.08 (1.39)	5.46 (1.54)	5.58 (2.04)	7.52 (3.35)	8.63 (4.23)	8.86 (4.26)	8.75 (3.94)
**c) Preventive migraine-specific medications**										
**All regions**										
Number of countries	40	39	40	46	53	59	62	63	63	62
Median (IQR^a^)	10.90 (0.00–184.29)	8.27 (0.00–132.58)	6.90 (0.00–128.22)	5.46 (0.00–111.86)	3.70 (0.00–58.50)	3.25 (0.03–61.82)	4.66 (0.11–48.48)	5.86 (0.18–52.98)	7.50 (0.22–69.93)	13.00 (0.18–98.55)
Unweighted mean (SE^b^)	170.43 (44.55)	174.05 (52.58)	167.15 (52.93)	142.76 (36.71)	117.70 (33.34)	110.36 (31.03)	106.56 (30.23)	107.60 (29.23)	102.40 (26.64)	106.95 (26.76)
Population-weighted mean (SE)^c^	68.50 (29.18)	67.56 (29.15)	63.42 (27.69)	64.35 (26.39)	60.90 (25.17)	56.39 (21.82)	52.70 (21.72)	63.70 (22.51)	67.60 (22.23)	71.47 (23.04)
**High-income countries/regions**										
Number of countries	24	22	23	30	35	40	40	40	40	40
Median (IQR)	17.69 (0.00–269.03)	12.89 (0.00–238.01)	14.10 (0.00–205.03)	12.12 (0.00–177.49)	7.32 (0.15–80.71)	7.43 (0.71–89.18)	8.60 (2.31–59.31)	9.41 (2.21–65.40)	12.31 (2.79–109.30)	21.92 (7.20–117.51)
Unweighted mean (SE)	217.04 (65.92)	224.38 (80.16)	216.20 (81.28)	167.19 (51.30)	135.19 (47.31)	120.44 (41.10)	123.06 (42.16)	121.91 (40.79)	113.51 (35.96)	124.40 (37.19)
Population-weighted mean (SE)	196.92 (66.70)	192.49 (67.04)	181.04 (63.91)	171.67 (57.27)	167.48 (56.43)	150.01 (46.64)	160.16 (48.33)	176.54 (47.86)	189.07 (45.26)	212.21 (47.30)
**Upper-middle-income countries/regions**										
Number of countries	9	10	10	9	10	10	12	13	13	13
Median (IQR)	0.07 (0.00–20.52)	0.06 (0.00–17.80)	0.04 (0.00–14.77)	0.01 (0.00–12.76)	0.00 (0.00–9.94)	0.05 (0.00–9.51)	0.12 (0.00–11.12)	0.13 (0.01–1.32)	0.13 (0.01–1.26)	0.15 (0.02–2.40)
Unweighted mean (SE)	51.18 (32.55)	52.07 (34.52)	53.13 (35.50)	80.62 (57.72)	55.82 (37.39)	52.62 (35.08)	50.23 (32.59)	50.10 (33.66)	48.97 (34.10)	49.07 (34.72)
Population-weighted mean (SE)	14.76 (21.06)	14.86 (22.29)	15.13 (23.00)	22.69 (36.87)	16.28 (24.54)	15.58 (23.25)	15.34 (21.78)	15.29 (22.53)	15.00 (22.59)	15.64 (22.97)
**Lower-middle-income countries/regions**										
Number of countries	7	7	7	7	8	9	10	10	10	9
Median (IQR)	28.22 (0.00–164.78)	27.36 (0.00–189.47)	26.40 (0.00–168.77)	4.08 (0.00–164.70)	5.53 (0.15–153.85)	3.22 (0.00–154.97)	5.13 (0.55–69.74)	4.27 (0.13–160.32)	9.41 (0.10–143.46)	3.93 (0.01–141.10)
Unweighted mean (SE)	150.93 (90.67)	143.51 (80.83)	130.60 (72.38)	132.05 (84.48)	134.38 (86.12)	150.88 (103.01)	119.47 (85.72)	130.86 (80.48)	132.77 (80.21)	118.18 (70.67)
Population-weighted mean (SE)	48.76 (46.93)	49.10 (45.20)	44.82 (41.09)	44.89 (43.89)	45.67 (44.86)	45.13 (47.40)	29.24 (35.84)	49.64 (46.40)	52.78 (49.45)	48.32 (44.34)
**d) Propranolol**										
**All regions**										
Number of countries	72	73	73	73	73	73	73	73	73	73
Median (IQR^a^)	1097.37 (337.79–1814.16)	1127.96 (366.50–1928.31)	1097.56 (377.63–1873.01)	1151.42 (361.64–1849.60)	1051.11 (339.11–1695.52)	1018.42 (312.65–1727.02)	1038.59 (382.60–1778.18)	1058.19 (359.52–1860.77)	1093.24 (362.98–1805.72)	1144.54 (345.05–1996.74)
Unweighted mean (SE^b^)	1302.35 (137.42)	1319.05 (141.87)	1338.38 (147.24)	1368.05 (153.89)	1369.46 (158.31)	1415.25 (169.04)	1468.18 (174.96)	1444.22 (177.59)	1475.10 (185.69)	1491.31 (193.29)
Population-weighted mean (SE)^c^	841.48 (112.66)	858.70 (119.73)	879.18 (124.55)	921.54 (131.16)	926.80 (135.24)	950.73 (143.96)	982.10 (149.07)	998.84 (154.27)	1006.96 (160.75)	1045.25 (170.06)
**High-income countries/regions**										
Number of countries	40	41	41	41	41	41	41	41	41	41
Median (IQR)	1295.55 (993.85–2187.31)	1328.09 (902.58–2305.63)	1314.06 (949.03–2358.29)	1313.40 (823.65–2377.71)	1347.55 (866.61–2500.16)	1354.20 (771.39–2456.16)	1379.36 (970.84–2595.28)	1379.33 (772.01–2679.77)	1445.14 (999.87–2672.42)	1373.01 (831.05–2834.64)
Unweighted mean (SE)	1737.54 (201.76)	1766.36 (207.12)	1804.70 (213.95)	1821.40 (227.72)	1823.30 (235.11)	1893.33 (250.92)	1983.91 (261.44)	1956.01 (267.50)	2030.80 (280.65)	2026.91 (295.60)
Population-weighted mean (SE)	1939.10 (173.39)	1993.67 (191.99)	2095.98 (201.30)	2171.64 (213.77)	2134.88 (236.94)	2228.52 (256.43)	2372.92 (267.57)	2441.26 (282.59)	2547.19 (301.33)	2660.50 (324.68)
**Upper-middle-income countries/regions**										
Number of countries	20	20	20	20	20	20	20	20	20	20
Median (IQR)	434.96 (243.70–1117.08)	356.82 (194.64–1176.90)	361.32 (221.18–1203.54)	347.63 (232.34–1224.99)	344.06 (242.70–1269.32)	307.94 (192.52–1302.81)	351.35 (229.64–1225.13)	332.56 (178.46–1189.16)	316.49 (176.91–1096.21)	319.69 (199.49–1233.26)
Unweighted mean (SE)	798.35 (172.83)	778.65 (181.67)	799.18 (195.86)	837.42 (202.42)	828.18 (210.69)	855.38 (232.67)	848.68 (223.36)	849.77 (226.94)	832.98 (227.70)	853.02 (237.84)
Population-weighted mean (SE)	521.32 (173.34)	498.12 (172.73)	507.19 (176.43)	528.62 (192.16)	543.09 (197.47)	562.85 (215.57)	556.56 (205.35)	564.98 (202.63)	571.54 (200.66)	574.10 (195.65)
**Lower-middle-income countries/regions**										
Number of countries	12	12	12	12	12	12	12	12	12	12
Median (IQR)	397.69 (278.46–1114.95)	406.69 (257.39–1145.50)	417.14 (214.53–1115.26)	422.91 (192.25–1252.36)	451.98 (198.97–1139.18)	472.24 (243.03–1091.27)	511.00 (230.36–1073.47)	499.92 (215.61–1089.97)	534.77 (151.02–1037.79)	550.82 (135.98–1174.64)
Unweighted mean (SE)	655.44 (157.71)	691.43 (176.68)	643.80 (169.37)	703.47 (184.86)	720.94 (191.74)	714.93 (191.38)	738.61 (192.16)	686.34 (177.12)	646.68 (156.02)	725.19 (186.88)
Population-weighted mean (SE)	537.00 (155.25)	581.22 (188.71)	568.64 (175.30)	619.16 (190.77)	642.78 (197.05)	633.32 (190.98)	646.49 (186.72)	643.43 (190.57)	595.13 (155.71)	628.89 (186.60)

^a^IQR: Interquartile range;

^b^SE: Standard error;

^c^The population-weighted mean was calculated by assigning weights proportional to each country’s population size for the respective year. The standard error of the weighted mean was derived from the unbiased weighted sample variance to account for the weights of different population sizes

### Acute MSM consumption trend

Acute MSMs accounted for the majority of overall MSM consumption (Table 1; Fig. 1). Across all countries/regions, the median consumption of acute MSMs increased from 52.65 (IQR: 5.90-240.27) in 2015 to 119.44 (IQR: 16.48-359.61) in 2024. Over the same period, the population-weighted mean rose from 83.12 (SE: 20.20) to 132.07 (SE: 30.39), demonstrating an upward trend that shifted from an APC of 3.3% (95% CI: 2.7% to 3.8%) during 2015–2018 to 6.5% (95% CI: 6.2% to 6.8%) from 2018 to 2024. Detailed annual trends in both unweighted average and median consumption of acute MSMs stratified by income-level groups are further illustrated in Fig. S2a-b. In HICs, the population-weighted mean correspondingly increasing from 296.21 (SE: 31.07) to 456.94 (SE: 38.14). This group exhibited APCs of 3.5% (95% CI: 2.2% to 4.7%) during 2015–2019 and 6.2% (95% CI: 5.5% to 6.8%) during 2019–2024. In UMICs, the population-weighted mean rose from 38.25 (SE: 30.44) to 72.81 (SE: 56.71), showing an overall APC of 7.6% (95% CI: 6.6% to 8.6%). Within LMICs, the population-weighted mean shifted from 3.69 (SE: 0.90) to 8.75 (SE: 3.94). The trend showed a substantial increase during the study period (APC: 11.4%, 95% CI: 9.7% to 13.1%). Detailed country-level data on acute MSM consumption during 2015–2024 were provided in Table S3.

**Fig. 2:**
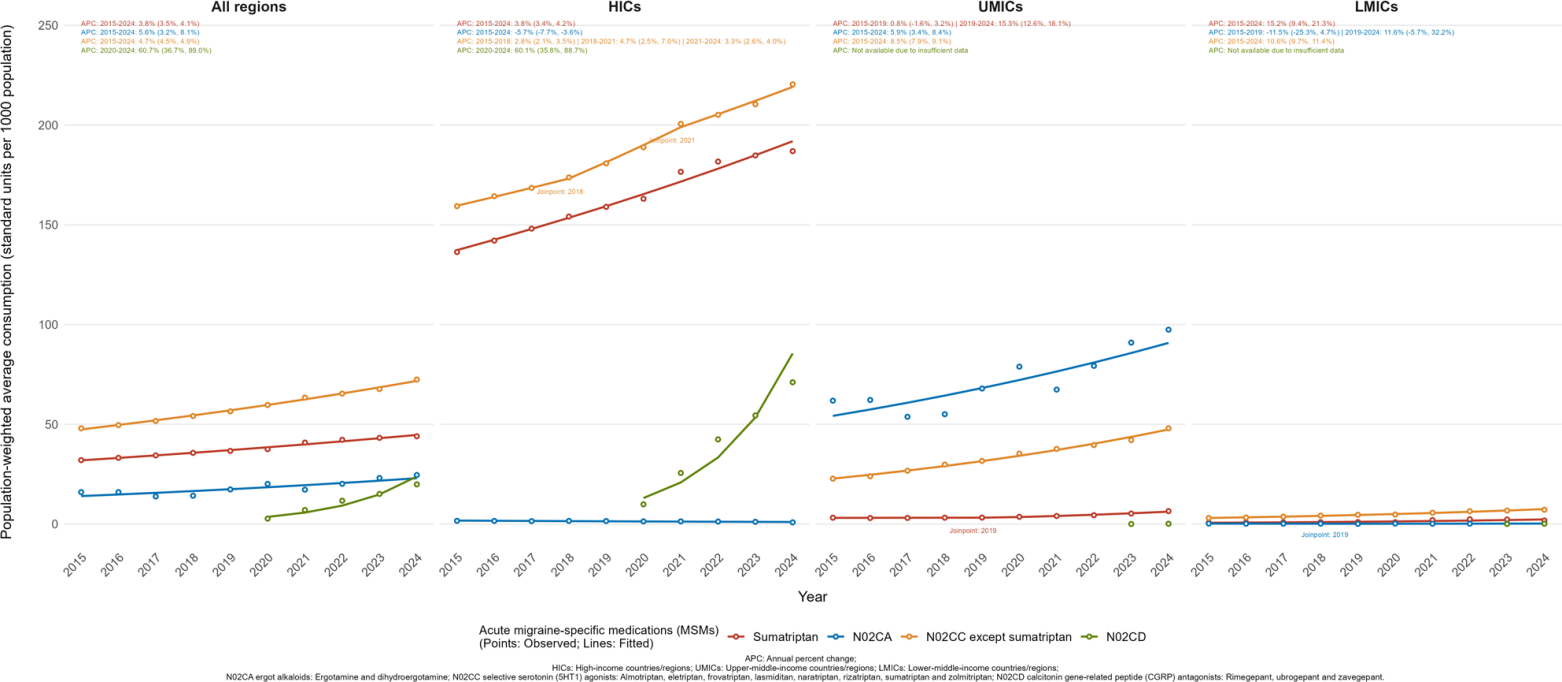
Trends in population-weighted average consumption of acute migraine-specific medications by income-level group, 2015–2024

Specifically, the median consumption of sumatriptan across all regions increased from 23.07 (IQR: 1.82–80.22) standard units per 1,000 population in 2015 to 39.90 (IQR: 5.26-154.33) in 2024, as shown in Table S4. In HICs, the median consumption of sumatriptan grew from 71.41 (IQR: 27.34-149.51) to 128.48 (IQR: 46.46-287.58), while the population-weighted mean correspondingly increased from 136.33 (SE: 18.81) to 186.91 (SE: 22.02). The trend in this group paralleled the overall pattern, recording an APC of 3.8% (95% CI: 3.4% to 4.2%) throughout the decade (Fig. 2). In UMICs, consumption levels were lower, changing from a median of 2.30 (IQR: 1.22–8.10) to 5.57 (IQR: 2.44–18.34). The population-weighted mean in this group increased from 3.18 (SE: 2.31) in 2015 to 6.46 (SE: 4.99) in 2024. Joinpoint analysis indicated a two-phase trajectory for UMICs, with a relatively flat initial period from 2015 to 2019 (APC: 0.8%, 95% CI: -1.6% to 3.2%), followed by an upward trend from 2019 to 2024 (APC: 15.3%, 95% CI: 12.6% to 18.1%). Within LMICs, sumatriptan consumption remained the lowest, with the median changing from 0.62 (IQR: 0.21–1.94) in 2015 to 1.18 (IQR: 0.09–3.33) in 2024, and the population-weighted mean shifting from 0.68 (SE: 0.17) to 1.75 (SE: 1.07). The trend in LMICs exhibited an overall APC of 15.2% (95% CI: 9.4% to 21.3%) from 2015 to 2024. Similar growth patterns were observed for other triptan and ditan agents excluding sumatriptan, as shown in Table S4 and Fig. 2.

Ergot alkaloids for acute migraine treatment (ergotamine and dihydroergotamine) were recorded in only a small number of countries, with ergotamine use reported mainly in Bolivia, Brazil, Germany, and Hungary, and dihydroergotamine recorded only in the United States and the Dominican Republic. In HICs, consumption remained low and the population-weighted mean decreased from 1.60 (SE: 4.36) in 2015 to 0.83 (SE: 2.39) by 2024 (APC: -5.7%, 95%CI: -7.7% to -3.6%). UMICs showed an overall increase annually by 5.9% (95%CI: 3.4%, 8.4%), reaching average 97.42 (SE: 75.50) in 2024, for which Brazil accounted for most of the recorded consumption. In LMICs, only Bolivia was recorded low and stable consumption of ergotamine over the decade (18.74 in 2015; 23.36 in 2024).

Consumption of CGRP inhibitors for acute migraine treatment (rimegepant, ubrogepant, and zavegepant) emerged recently during the study period. Initial records appeared only in the United States in 2020, rapidly expanding to 46 countries/regions by 2024. Across these countries/regions, the population-weighted mean consumption increased from 2.71 (SE: 1.35) standard units per 1,000 population in 2020 to 19.87 (SE: 9.15) in 2024 (Table S4). As shown in Fig. 2, the overall trend demonstrated a steep upward trend with an APC of 60.7% (95%CI: 36.7% to 89.0%). These medications were first introduced and predominantly utilized in HICs, where availability expanded to 35 countries/regions by 2024. In this group, the population-weighted mean surged from 9.86 (SE: 2.40) in 2020 to 71.06 (SE: 15.87) in 2024 (APC: 60.1%, 95%CI: 35.8% to 88.7%). In MICs, consumption was not recorded until 2023 and remained minimal, with only rimegepant reported. By 2024, these agents were recorded in 8 UMICs and 3 LMICs. The population-weighted means in 2024 were low, recorded at 0.14 (SE: 0.14) in UMICs and 0.16 (SE: 0.04) in LMICs.

### Preventive MSM consumption trend

Not all countries/regions in this study recorded preventive MSM consumption consistently over the 10-year period. As detailed in Table 1 and Table S3, the population-weighted mean consumption rose from 68.50 (SE: 29.18) standard units per 1,000 population in 2015 to 71.47 (SE: 23.04) in 2024. Overall, Fig. 1 showed that the population-weighted mean consumption declined between 2015 and 2021 (APC: -3.8%, 95% CI: -5.4% to -2.1%), followed by a significant increase thereafter (APC: 9.8%, 95% CI: 6.4% to 13.4%). Detailed annual trends in both unweighted average and median consumption of preventive MSMs stratified by income-level groups are further illustrated in Fig. S3a-b. In HICs, the population-weighted mean increased from 196.92 (SE: 66.70) to 212.21 (SE: 47.30) and the median from 17.69 (IQR: 0.00-269.03) to 21.92 (IQR: 7.20-117.51), with a decline through 2020 (APC: -5.1%, 95% CI: -6.0% to -4.2%) followed by an increase thereafter (APC: 9.6%, 95% CI: 7.6% to 11.5%). At the country level, consumption increased markedly in the United States (74.95 in 2015 to 321.31 in 2024) and Canada (25.55 to 281.30), whereas Ireland decreased from 1614.15 to 73.53 over the same period. In UMICs, preventive MSM consumption remained low (mean: 14.76 to 15.64; median: 0.07 to 0.15), with a non-significant increase up to 2018 (APC: 9.1%, 95% CI: -7.3% to 28.4%) followed by a decline thereafter (APC: -3.8%, 95% CI: -7.9% to -0.5%). Most UMICs reported negligible consumption, with notable exceptions in Algeria (438.80 to 466.60) and South Africa (183.49 to 185.86). In LMICs, the population-weighted mean remained relatively stable (48.76 to 48.32), whereas the median declined from 28.22 (IQR: 0.00-164.78) to 3.93 (IQR: 0.01–141.10). At the country level, consumption declined to zero in Morocco (98.96 to 0), whereas Tunisia remained consistently high despite a decrease (929.58 to 699.33) over the study period.

**Fig. 3:**
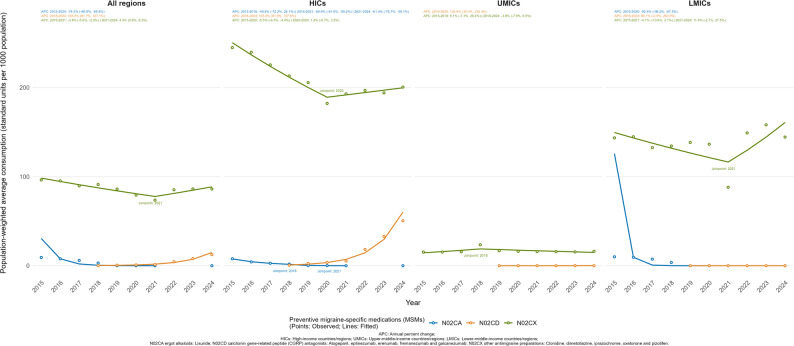
Trends in population-weighted average consumption of preventive migraine-specific medications by income-level group, 2015–2024

Lisuride, as the ergot alkaloid for migraine prevention, was recorded in only five countries over the study period, including Egypt, France, Germany, New Zealand, and Pakistan. In HICs, median consumption decreased from 10.90 (IQR: 6.75–12.43) in 2015 to less than 0.01 in 2024 (Table S5 and Fig. 3). No consumption was recorded in UMICs, while in LMICs consumption declined from 7.28 (IQR: 3.64–10.92) in 2015 to zero by 2022.

Preventive CGRP inhibitors were not recorded before 2018 and were reported in 16 countries/regions in 2018, increasing to 57 countries/regions by 2024, as detailed in Table S5. Among these countries/regions, the median consumption increased from near-zero in 2018 to 1.30 (IQR: 0.09–8.99) in 2024, while the population-weighted mean rose from 0.15 (SE: 0.08) to 12.42 (SE: 6.15). Preventive CGRP inhibitors were rapidly adopted in HICs (APC: 103.5%, 95%CI: 81.9% to 127.6%, Fig. 3), where the population-weighted mean consumption increased from 0.62 (SE: 0.17) in 2018 to 50.51 (SE: 12.56) in 2024. In MICs, population-weighted mean consumption remained low, reaching only 0.04 (SE: 0.03) in UMICs and less than 0.01 in LMICs in 2024, although UMICs showed a significant increase (APC: 136.9%, 95% CI: 30.4% to 330.4%).

Other antimigraine preparations under ATC code N02CX accounted for the largest share of preventive MSM consumption (Fig. 3). As shown in Table S5, in HICs, the population-weighted mean consumption decreased from 244.87 (SE: 93.30) standard units per 1,000 population in 2015 to 182.20 (SE: 65.12) in 2020, marked by a significant negative trend (APC: -5.5%, 95% CI: -6.5% to -4.4%). Subsequently, the trend stabilized, showing a slight non-significant increase to 200.54 (SE: 63.29) by 2024 (APC: 1.4%, 95% CI: -0.7% to 3.5%). In MICs, consumption patterns also showed distinct multiphase trends. Notably, UMICs consistently recorded the lowest consumption levels among all income groups. In UMICs, the population-weighted mean experienced a non-significant initial increase from 15.29 (SE: 25.89) in 2015 to 23.53 (SE: 45.33) in 2018 (APC: 9.1%, 95% CI: -7.3% to 28.4%), before declining to 16.19 (SE: 28.26) by 2024 (APC: -3.8%, 95% CI: -7.9% to 0.5%). By contrast, LMICs maintained substantially higher baseline consumption than UMICs. In LMICs, the population-weighted mean changed from 143.50 (SE: 56.76) in 2015 to 144.41 (SE: 53.60) in 2024 (2015–2021 APC: -4.1%, 95% CI: -10.8% to 3.1%; 2021–2024 APC: 11.4%, 95% CI: -2.7% to 27.5%).

### Propranolol consumption trend

Overall, the consumption of propranolol maintained a vast and relatively stable baseline compared to other preventive agents. As shown in Fig. S4a-c and Table S3, in HICs, the population-weighted mean consumption grew from 1,939.10 (SE: 173.39) to 2,660.50 (SE: 324.68) by 2024, supported by a significant upward trend (2015–2020 APC: 2.8%, 95% CI: 1.8% to 3.8%; 2020–2024 APC: 4.3%, 95% CI: 3.3% to 5.4%). Despite the generally high consumption in this income group, consumption was exceptionally low in the Czech Republic (0.00 in 2015 to 4.40 in 2024) and the Slovak Republic (0.49 in 2015 to 11.56 in 2024) throughout the study period. In UMICs, the population-weighted mean moderately increased from 521.32 (SE: 173.34) in 2015 to 574.10 (SE: 195.65) in 2024 (APC: 1.6%, 95% CI: 1.1% to 2.0%). At the national level, Kazakhstan experienced a sharp decline in consumption from 858.78 in 2015 to 5.62 in 2024. Conversely, in LMICs, the population-weighted mean rose from 537.00 (SE: 155.25) in 2015 to 628.89 (SE: 186.60) in 2024, characterized by an initial increase from 2015 to 2019 (APC: 4.3%, 95% CI: 2.4% to 6.3%) followed by a slight decline through 2024 (APC: -1.0%, 95% CI: -2.8% to 0.9%). Despite this overall growth, several countries recorded persistently low and declining consumption, notably Bolivia (47.42 to 11.56), the Philippines (117.67 to 44.32), and Vietnam (148.90 to 67.02) from 2015 to 2024.

### Changes in non-CGRP MSM and propranolol consumption following CGRP introduction and COVID-19 onset

**Fig. 4:**
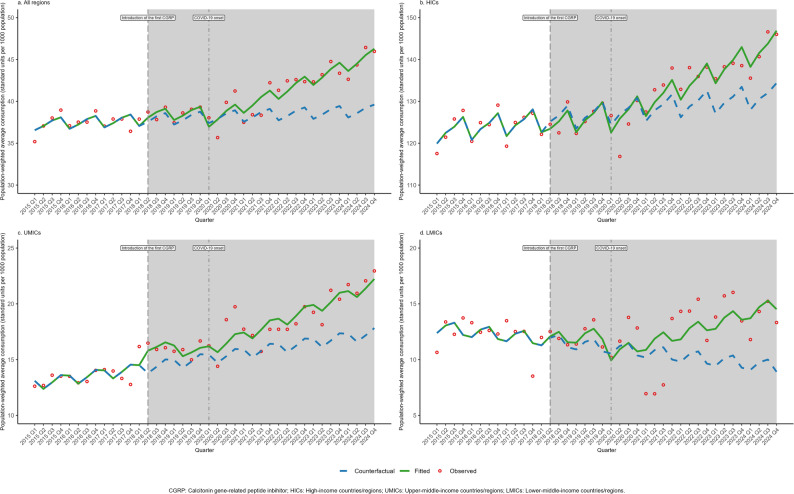
Impact of introduction of the first calcitonin gene-related peptide inhibitor and COVID-19 onset on migraine-specific medication consumption stratified by income-level group, 2015–2024

**Fig. 5:**
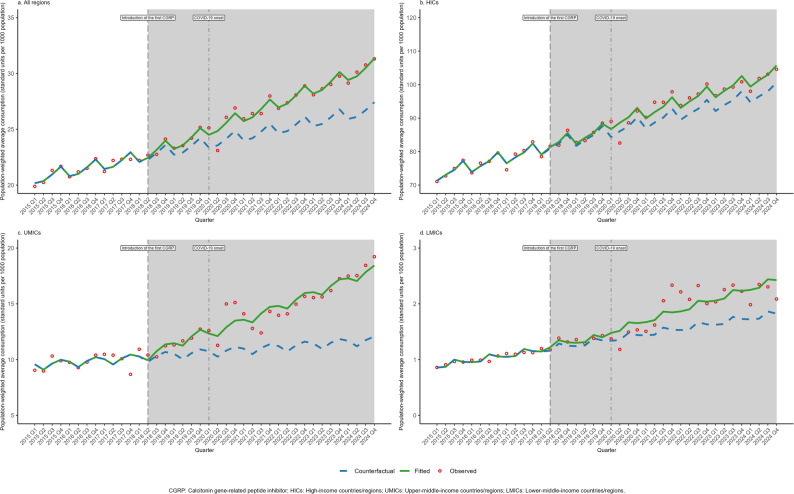
Impact of introduction of the first calcitonin gene-related peptide inhibitor and COVID-19 onset on acute migraine-specific medication consumption stratified by income-level group, 2015–2024

**Fig. 6:**
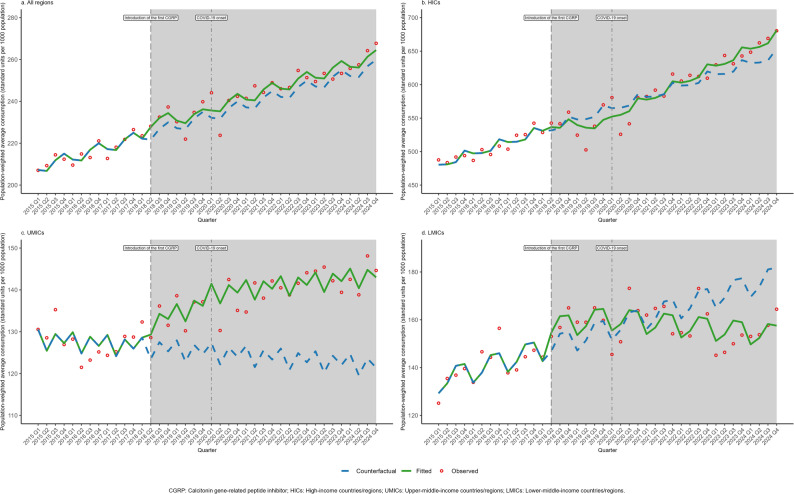
Impact of introduction of the first calcitonin gene-related peptide inhibitor and COVID-19 onset on propranolol consumption stratified by income-level group, 2015–2024

In this exploratory analysis of population-weighted average consumption following key events (Table 2; Figs. 4, 5, 6), changes in consumption (level changes) and growth rates (slope changes) are expressed in standard units per 1,000 population and standard units per 1,000 population per quarter, respectively. In the patterns observed after the introduction of the first CGRP inhibitor, consumption of non-CGRP MSMs showed varying shifts across income groups. HICs recorded a significant immediate decline in consumption (-1.990, 95% CI: -3.627 to -0.352). Conversely, UMICs showed an initial increase in consumption (2.258, 95% CI: 1.466 to 3.049), which was accompanied by a subsequent deceleration in the quarterly growth rate (-0.241, 95% CI: -0.390 to -0.092). For acute non-CGRP MSMs, the overall trend across all regions exhibited an accelerated growth rate (0.119, 95% CI: 0.074 to 0.164). Furthermore, propranolol exhibited significant immediate increases in consumption across all regions (6.837, 95% CI: 4.152 to 9.522), particularly pronounced in UMICs and LMICs.

**Table 2:** Changes in population-weighted consumption of migraine-specific medications and propranolol after introduction of the first calcitonin gene-related peptide inhibitor and COVID-19 onset

	Baseline level (95% CI)	*P*-value	Baseline slope (95% CI)	*P*-value	Level change after introduction of the first CGRP inhibitor (95% CI)	*P*-value	Slope change after introduction of the first CGRP inhibitor (95% CI)	*P*-value	Level change after COVID-19 (95% CI)	*P*-value	Slope change after COVID-19 (95% CI)	*P*-value
**Population-weighted average MSM consumption except any CGRP inhibitors** ^a^												
All regions	36.515 (35.451, 37.579)	< 0.001*	0.042 (-0.060, 0.144)	0.416	0.475 (-0.435, 1.384)	0.306	0.016 (-0.128, 0.160)	0.826	-1.404 (-2.050, -0.759)	< 0.001*	0.358 (0.234, 0.483)	< 0.001*
HICs	119.689 (117.393, 121.985)	< 0.001*	0.224 (0.049, 0.399)	0.012*	-1.990 (-3.627, -0.352)	< 0.017*	0.277 (-0.009, 0.563)	0.058	-2.549 (-6.118, 1.020)	0.162	0.479 (0.060, 0.898)	< 0.025*
UMICs	12.982 (12.521, 13.444)	< 0.001*	0.117 (0.043, 0.191)	0.002*	2.258 (1.466, 3.049)	< 0.001*	-0.241 (-0.390, -0.092)	< 0.002*	-0.010 (-1.423, 1.403)	0.989	0.433 (0.301, 0.565)	< 0.001*
LMICs	12.475 (10.761, 14.189)	< 0.001*	-0.092 (-0.246, 0.063)	0.246	-0.038 (-1.353, 1.277)	0.955	0.161 (-0.071, 0.393)	0.173	-2.045 (-4.195, 0.106)	0.062	0.167 (-0.074, 0.408)	0.175
**Population-weighted average acute MSM consumption except any CGRP inhibitors**												
All regions	20.004 (19.702, 20.307)	< 0.001*	0.160 (0.133, 0.187)	< 0.001*	0.046 (-0.246, 0.339)	0.756	0.119 (0.074, 0.164)	< 0.001*	0.112 (-0.205, 0.428)	0.490	0.028 (-0.022, 0.078)	0.271
HICs	70.636 (69.571, 71.701)	< 0.001*	0.651 (0.601, 0.702)	< 0.001*	0.418 (-0.380, 1.216)	0.304	0.043 (-0.064, 0.151)	0.430	1.532 (-0.788, 3.852)	0.196	0.100 (-0.134, 0.333)	0.402
UMICs	9.526 (9.034, 10.019)	< 0.001*	0.058 (-0.002, 0.118)	0.056	-0.130 (-0.771, 0.511)	0.691	0.271 (0.178, 0.363)	< 0.001*	-0.423 (-1.674, 0.828)	0.508	-0.020 (-0.107, 0.066)	0.643
LMICs	0.834 (0.769, 0.900)	< 0.001*	0.024 (0.021, 0.027)	< 0.001*	0.063 (-0.010, 0.137)	0.092	-0.001 (-0.014, 0.012)	0.893	0.060 (-0.261, 0.380)	0.716	0.025 (-0.004, 0.054)	0.094
**Population-weighted average propranolol consumption**												
All regions	205.953 (203.062, 208.845)	< 0.001*	1.248 (1.012, 1.484)	< 0.001*	6.837 (4.152, 9.522)	< 0.001*	-0.798 (-1.659, 0.063)	0.069	2.178 (-2.443, 6.798)	0.356	0.857 (0.010, 1.703)	0.047*
HICs	476.221 (464.274, 488.168)	< 0.001*	4.224 (3.243, 5.204)	< 0.001*	9.441 (-8.079, 26.962)	0.291	-4.442 (-9.620, 0.736)	0.093	6.903 (-18.310, 32.116)	0.592	6.558 (1.586, 11.531)	0.010*
UMICs	130.709 (126.185, 135.234)	< 0.001*	-0.163 (-0.687, 0.361)	0.542	4.885 (0.706, 9.063)	0.022*	0.949 (0.359, 1.538)	0.002*	2.254 (-1.910, 6.418)	0.289	-0.558 (-0.924, -0.193)	0.003*
LMICs	128.127 (123.426, 132.828)	< 0.001*	1.120 (0.611, 1.629)	< 0.001*	8.198 (2.153, 14.243)	0.008*	-0.442 (-1.802, 0.919)	0.524	0.233 (-5.854, 6.320)	0.940	-1.040 (-2.538, 0.459)	0.174

Note on units: Estimates for baseline level and level change are expressed as standard units per 1000 population; Estimates for baseline slope and slope change represent the rate of change, expressed as standard units per 1000 population per quarter

a MSM: Migraine-specific medication; CGRP inhibitor: calcitonin gene-related peptide inhibitor; Population-weighted average MSM consumption includes both acute and preventive MSMs except any CGRP inhibitors;

* P-values marked with an asterisk indicate statistical significance at *p* < 0.05;

HICs: high-income countries/regions; UMICs: upper-middle-income countries/regions; LMICs: lower-middle-income countries/regions; CI: confidence interval

The onset of the COVID-19 pandemic was temporally associated with an immediate decline in overall consumption across all regions for non-CGRP MSMs (-1.404, 95% CI: -2.050 to -0.759), followed by a significant coinciding upward shift in the long-term growth rate (0.358, 95% CI: 0.234 to 0.483). This post-pandemic acceleration was statistically significant in both HICs (0.479, 95% CI: 0.060 to 0.898) and UMICs (0.433, 95% CI: 0.301 to 0.565). Notably, no statistically significant changes in immediate consumption or growth rates were observed for acute non-CGRP MSMs across any income group after the pandemic onset (all *p* > 0.05). For propranolol, the post-pandemic growth rate accelerated in HICs (6.558, 95% CI: 1.586 to 11.531) but decelerated in UMICs (-0.558, 95% CI: -0.924 to -0.193). Detailed country-level changes in consumption following these events are presented in Fig. S5-S7.

## Discussion

In this multinational analysis of 73 countries/regions from 2015 to 2024, we observed a persistent disparity in MSM consumption, with growth largely concentrated in HICs and disproportionately low population-adjusted levels in UMICs and LMICs. Within this overall pattern, acute MSMs, mainly driven by triptans, accounted for most of the consumption and showed accelerating growth in HICs and UMICs, whereas this acute-dominant pattern was not evident in LMICs. While preventive MSM consumption experienced an initial decline followed by a slight rebound, propranolol, the only preventive medicine included in the WHO EML, maintained stable and substantial consumption in most of the included countries/regions. In our exploratory analysis, the rapid adoption of CGRP inhibitors in HICs temporally coincided with an immediate decline in non-CGRP MSM consumption, whereas their consumption in MICs remained minimal. The onset of COVID-19 pandemic was associated with a general short-term drop in overall non-CGRP MSM consumption, followed by an accelerated long-term growth trend in HICs and UMICs. These divergent post-event patterns highlight how macro-level shifts may have further widened existing cross-national inequalities.

The notable disparity in overall MSM consumption and the low utilization of acute MSMs in MICs, especially in the LMICs, reflect a structural barrier within healthcare system. Specifically, accurate migraine diagnosis depends largely on detailed clinical history rather than objective biomarkers [12]. This diagnostic reality poses two major clinical challenges in regions lacking trained headache specialists. First, a substantial portion of patients remain completely undiagnosed due to the severe shortage of accessible neurological care [34]. Second, patients who do seek help in primary care clinics could often be misdiagnosed by general practitioners as having routine tension type or sinus headaches [35, 36]. Consequently, both undiagnosed and misdiagnosed patients are managed entirely outside formal neurological care pathways where established clinical criteria would normally trigger targeted prescribing. Without a formal diagnosis, patients with migraine cannot access MSMs and instead heavily rely on inexpensive broad-spectrum analgesics including over the counter NASIDs and paracetamol [37]. Even when a formal clinical diagnosis is established, access to appropriate treatment may still be constrained. The relatively high cost of acute MSMs and the inconsistent inclusion of essential agents, such as triptans, in national formularies across many MICs further limit their use [38, 39]. These diagnostic and economic barriers help explain the low population-level consumption of acute MSMs observed in these regions.

As for preventive pharmacotherapy, propranolol consumption remains difficult to interpret in our study because it encompasses broad cardiovascular and other indications, making it impossible to quantify its migraine-specific prophylactic use. Nevertheless, its widespread and consistent consumption suggests that it is broadly accessible across all income groups. However, such availability may not translate to equitable migraine care, particularly in healthcare systems where its use is largely confined to cardiovascular indications due to resource prioritization [34, 40]. Furthermore, while older migraine-specific preventives like lisuride showed a consistent decline, other antimigraine preparations (ATC code N02CX) continued to represent the largest share of preventive consumption across most regions despite varying trend directions between income groups. Our study observed that while preventive MSM consumption has increased since 2021, primarily driven by the rapid adoption of CGRP inhibitors in HICs, consumption of these novel agents remains negligible in MICs. In clinical practice, strict step-therapy protocols, insurance authorization mandates, and prohibitive out of pocket costs dictate that these advanced interventions remain restricted to a highly selected refractory patient subset, even within the most resourceful healthcare systems [41]. Therefore, the rapid market growth of CGRP inhibitors may give the impression of progress while underlying gaps in preventive care persist. Without corresponding structural pricing reforms, these advanced agents may exacerbate the global treatment divide rather than resolve the profound underutilization of migraine-specific preventive pharmacotherapy.

The overall increase in MSM consumption over the past decade likely reflects key diagnostic and therapeutic advancements. First, the publication of the International Classification of Headache Disorders, 3rd edition (ICHD-3) in 2018 provided standardized and more universally applicable diagnostic criteria [1], which may have improved global detection and diagnosis rates in routine clinical practice. Concurrently, updated treatment guidelines may have increasingly discouraged the overuse of NSAIDs and the use of oral ergot alkaloids and opioids [12, 42], advocating instead for timely initial of preventive therapies for eligible patients [43]. Together, these shifts toward standardized diagnosis and targeted treatment likely contributed to the accelerated MSM consumption observed in well-resourced healthcare systems. However, the substantial cross-national differences in MSM consumption, together with the particularly low levels observed in MICs, reflect a systemic misalignment in international healthcare prioritization. Although migraine ranks among the leading global causes of DALYs [9], its non-fatal nature often results in it being overlooked relative to conditions with higher mortality [44]. In practice, both national health systems and international funding agencies tend to prioritize life-threatening diseases, leaving severe but non-fatal neurological disorders underfunded. This pervasive mortality bias likely contributes to the limited inclusion of MSMs in national formularies and the continued reliance on non-specific analgesics. To bridge this cross-national pharmaceutical divide, policymakers are required to better align drug procurement and formulary decisions with the actual disability burden of migraine, rather than relying on mortality-centric resource allocation.

To the best of our knowledge, this is the first cross-national investigation to directly compare the standardized consumption of MSMs across different economic settings. A key strength of this work lies in its utilization of a standardized global database, which offers a macro-level view of real-world dispensing patterns. Unlike studies based on regional prescription sampling or insurance claims, our analysis captured systemic disparities in migraine-specific pharmacotherapies and allowed a more consistent comparison of MSM penetration across countries over time. However, several limitations should be considered. First, as an ecological analysis based on aggregated data, we were unable to adjust for important patient-level factors such as disease severity, socioeconomic status, or local insurance coverage. As a result, the findings cannot be used to infer causal relationships at the individual level. Second, sales data do not capture the substantial use of over-the-counter analgesics. This is particularly relevant in MICs, where nonprescription treatments are widely used for acute relief, and may lead to an underestimation of the overall burden of self-medication. Third, as a study based on absolute sales volume, we lack the matched epidemiological denominators required to calculate definitive clinical underutilization rates. Although our standard unit metrics demonstrate profound cross-national disparities in medication dispensation, they cannot be directly translated into individual treatment gaps without concurrent and standardized global prevalence data. Fourth, the ITS analysis is constrained by the short interval between the introduction of CGRP inhibitors in 2018 Q2 and the onset of the COVID-19 pandemic in early 2020. Because this intermediate phase comprises only seven quarters, the natural adoption trend of CGRP inhibitors was likely truncated. Given the widespread disruption of healthcare systems during the pandemic [45], it remains difficult to separate true adoption trends from pandemic-related effects. Therefore, the long-term impact of CGRP inhibitors observed in our study must be interpreted with appropriate caution and contextualized within the COVID-19 pandemic. Finally, our study included 73 countries but did not cover low-income countries. Moreover, HICs (*n* = 41) accounted for a larger share of the included countries, compared with UMICs (*n* = 20) and LMICs (*n* = 12). This imbalance may bias the overall estimates toward patterns observed in HICs and limit the generalizability of our findings for resource-limited regions.

In conclusion, this decade-long multinational analysis highlights persistent disparities in access to MSMs, with overall consumption remaining overwhelmingly concentrated in HICs. The introduction of CGRP inhibitors has not reduced these inequalities and may instead reflect more restricted access to advanced migraine-specific preventive pharmacotherapy. Addressing these disparities will require health systems to better recognize the burden of migraine and improve the affordability and accessibility of migraine-specific pharmacotherapies.

## Supplementary Information

Below is the link to the electronic supplementary material.

Supplementary Material 1

## Data Availability

Data is not available as the data custodian has not given permission for data sharing.
