# Supplementary material for: Trends in migraine-specific medication consumption: a multinational analysis of 73 countries based on IQVIA-MIDAS database, 2015 to 2024
Source: J Headache Pain. 2026 Apr 15;27(1):145. doi: 10.1186/s10194-026-02363-6 (PMC13191992; doi:10.1186/s10194-026-02363-6)

Supplementary materials

Supplementary Methods. Details of interrupted time series model

Table S1. List of countries/regions included

Table S2. List of migraine-specific medications included

Table S3. Country-level consumption of migraine-specific medications, 2015-2024

Table S4. Consumption of acute migraine-specific medications by income-level group, 2015-2024

Table S5. Consumption of preventive migraine-specific medications by income-level group, 2015-2024

Figure S1a. Trends in unweighted average consumption of migraine-specific medications by income-level group, 2015-2024

Figure S1b. Trends in median consumption of migraine-specific medications by income-level group, 2015-2024

Figure S2a. Trends in unweighted average consumption of acute migraine-specific medications by income-level group, 2015-2024

Figure S2b. Trends in median consumption of acute migraine-specific medications by income-level group, 2015-2024

Figure S3a. Trends in unweighted average consumption of preventive migraine-specific medications by income-level group, 2015-2024

Figure S3b. Trends in median consumption of preventive migraine-specific medications by income-level group, 2015-2024

Figure S4a. Trends in population-weighted average consumption of propranolol by income-level group, 2015-2024

Figure S4b. Trends in unweighted average consumption of propranolol by income-level group, 2015-2024

Figure S4c. Trends in median consumption of propranolol by income-level group, 2015-2024

Figure S5. Impact of introduction of the first calcitonin gene-related peptide inhibitor and COVID-19 onset on migraine-specific medication consumption stratified by country, 2015-2024

Figure S6. Impact of introduction of the first calcitonin gene-related peptide inhibitor and COVID-19 onset on acute migraine-specific medication consumption stratified by country, 2015-2024

Figure S7. Impact of introduction of the first calcitonin gene-related peptide inhibitor and COVID-19 onset on propranolol consumption stratified by country, 2015-2024

Supplementary Methods. Details of interrupted time series model

The model used to estimate short-term and long-term effect of two major events on the consumption of migraine-specific medications was as follows:

$Y_{t}= \beta_{0}+ \beta_{1}\times t+ \beta_{2}\times{CGRP}_{t}+\beta_{3}\times{time after CGRP}_{t}+ \beta_{4}\times{COVID19}_{t}+\beta_{5}\times{time after COVID19}_{t}+\beta_{6}\times{quarter}_{t}+\epsilon_{t}$

Where:

- $Y_{t}$ is the sales volume at time t;
- $t$ is the number of quarters since 2015 Q1;
- ${CGRP}_{t}$ is the binary variable equal to 0 when calcitonin gene-related peptide (CGRP) inhibitors have not approved and marketed, 1 otherwise;
- ${time after CGRP}_{t}$ is the number of quarters since the introduction of CGRP inhibitors, which is expressed as ${time after CGRP}_{t}=max(0, t-t^{'}+1)$ where $t^{'}$ is the quarter when the CGRP inhibitors first approved and marketed;
- ${COVID19}_{t}$ is the binary variable equal to 0 when the COVID-19 has not occurred, 1 otherwise;
- ${time after COVID19}_{t}$ is the number of quarters since the onset of COVID-19, which is expressed as ${time after COVID19}_{t}=max(0, t-t^{'}+1)$ where $t^{'}$ is the quarter when the COVID-19 pandemic first occurred;
- ${quarter}_{t}$ is a factor variable with value of Q1, Q2, Q3 or Q4 to account for potential seasonality;
- $\beta_{0}$ is the baseline level of $Y_{t}$ where $t=0$ (intercept);
- $\beta_{1}$ is the change of $Y_{t}$ over time prior to intervention (slope);
- $\beta_{2}$ is the intercept offset for the first quarter of the introduction of CGRP inhibitors and after;
- $\beta_{3}$ is the slope offset for the first quarter of the introduction of CGRP inhibitors and after;
- $\beta_{4}$ is the intercept offset for the first quarter of the COVID-19 onset and after;
- $\beta_{5}$ is the slope offset for the first quarter of the COVID-19 onset and after;
- $\beta_{6}$ is the seasonal offset;
- $\epsilon_{t}$ is the random error.

The Newey-West method was used to adjust time series autocorrelation.

Table S1. List of countries/regions included

| Income-level group | Country/Region |
| --- | --- |
| High-income countries/regions (41) | Australia |
|  | Austria |
|  | Belgium |
|  | Bulgaria |
|  | Canada |
|  | Chile |
|  | Croatia |
|  | Czech Republic |
|  | Estonia |
|  | Finland |
|  | France |
|  | Germany |
|  | Greece |
|  | Hong Kong, China |
|  | Hungary |
|  | Ireland |
|  | Italy |
|  | Japan |
|  | South Korea |
|  | Kuwait |
|  | Latvia |
|  | Lithuania |
|  | Luxembourg |
|  | Netherlands |
|  | New Zealand |
|  | Norway |
|  | Poland |
|  | Portugal |
|  | Romania |
|  | Russian Federation |
|  | Saudi Arabia |
|  | Singapore |
|  | Slovak Republic |
|  | Slovenia |
|  | Spain |
|  | Sweden |
|  | Switzerland |
|  | United Arab Emirates |
|  | United Kingdom |
|  | United States |
|  | Uruguay |
| Upper-middle-income countries/regions (20) | Algeria |
|  | Argentina |
|  | Belarus |
|  | Bosnia and Herzegovina |
|  | Brazil |
|  | China |
|  | Colombia |
|  | Dominican Republic |
|  | Ecuador |
|  | Indonesia |
|  | Kazakhstan |
|  | Malaysia |
|  | Mexico |
|  | Paraguay |
|  | Peru |
|  | Serbia |
|  | South Africa |
|  | Thailand |
|  | Turkey |
|  | Venezuela |
| Lower-middle-income countries/regions (12) | Bangladesh |
|  | Bolivia |
|  | Egypt |
|  | India |
|  | Jordan |
|  | Lebanon |
|  | Morocco |
|  | Pakistan |
|  | Philippines |
|  | Sri Lanka |
|  | Tunisia |
|  | Vietnam |

Table S2. List of migraine-specific medications included

| WHO ATC code | Generic name | Clinical use (acute vs preventive) |
| --- | --- | --- |
| N02CA01 | dihydroergotamine | acute |
| N02CA02 | ergotamine | acute |
| N02CA07 | lisuride | preventive |
| N02CC01 | sumatriptan | acute |
| N02CC02 | naratriptan | acute |
| N02CC03 | zolmitriptan | acute |
| N02CC04 | rizatriptan | acute |
| N02CC05 | almotriptan | acute |
| N02CC06 | eletriptan | acute |
| N02CC07 | frovatriptan | acute |
| N02CC08 | lasmiditan | acute |
| N02CD01 | erenumab | preventive |
| N02CD02 | galcanezumab | preventive |
| N02CD03 | fremanezumab | preventive |
| N02CD04 | ubrogepant | acute |
| N02CD05 | eptinezumab | preventive |
| N02CD06 | rimegepant | acute |
| N02CD07 | atogepant | preventive |
| N02CD08 | zavegepant | acute |
| N02CX01 | pizotifen | preventive |
| N02CX02 | clonidine | preventive |
| N02CX03 | iprazochrome | preventive |
| N02CX05 | dimetotiazine | preventive |
| N02CX06 | oxetorone | preventive |

Table S3. Country-level consumption of migraine-specific medications, 2015-2024

**a) All migraine-specific medications**

|  | Consumption (standard units per 1,000 population) | | | | | | | | | |
| --- | --- | --- | --- | --- | --- | --- | --- | --- | --- | --- |
| Country/Region | 2015 | 2016 | 2017 | 2018 | 2019 | 2020 | 2021 | 2022 | 2023 | 2024 |
| **High-income countries/regions** | | | | | | | | | | |
| Australia | 782.31 | 827.12 | 801.76 | 853.42 | 878.94 | 906.93 | 936.57 | 936.15 | 924.62 | 952.49 |
| Austria | 240.27 | 246.05 | 247.90 | 255.17 | 254.06 | 253.65 | 270.28 | 282.20 | 283.83 | 299.30 |
| Belgium | 764.55 | 776.55 | 769.30 | 774.13 | 475.00 | 409.6 | 447.36 | 440.76 | 477.67 | 497.05 |
| Bulgaria | 27.10 | 30.11 | 35.24 | 39.86 | 42.33 | 41.98 | 45.67 | 51.94 | 60.14 | 63.75 |
| Canada | 458.75 | 467.7 | 459.97 | 451.79 | 467.57 | 482.96 | 489.88 | 518.34 | 656.77 | 766.93 |
| Chile | 136.19 | 145.42 | 151.03 | 157.18 | 167.96 | 184.37 | 230.44 | 229.54 | 227.61 | 244.91 |
| Croatia | 95.91 | 100.06 | 104.14 | 113.85 | 119.47 | 127.84 | 137.00 | 146.72 | 168.03 | 180.63 |
| Czech Republic | 259.39 | 256.70 | 279.82 | 283.25 | 298.00 | 301.87 | 311.07 | 327.97 | 313.81 | 309.75 |
| Estonia | 139.76 | 153.30 | 161.27 | 193.38 | 207.24 | 240.12 | 234.43 | 332.54 | 334.74 | 378.13 |
| Finland | 585.69 | 607.93 | 620.87 | 687.36 | 704.18 | 698.54 | 739.63 | 717.91 | 793.34 | 847.13 |
| France | 917.63 | 929.13 | 885.18 | 870.41 | 883.68 | 861.58 | 900.65 | 888.93 | 875.12 | 755.63 |
| Germany | 357.51 | 349.10 | 354.29 | 355.70 | 371.80 | 375.78 | 387.31 | 392.21 | 407.64 | 418.62 |
| Greece | 186.28 | 197.95 | 210.02 | 231.35 | 244.21 | 255.37 | 290.18 | 300.72 | 328.4 | 359.76 |
| Hong Kong, China | 60.96 | 52.21 | 57.81 | 58.15 | 55.99 | 66.31 | 71.33 | 72.72 | 76.50 | 74.96 |
| Hungary | 487.6 | 481.03 | 469.12 | 481.22 | 434.73 | 412.42 | 451.09 | 460.49 | 428.44 | 404.96 |
| Ireland | 1795.62 | 2767.93 | 2941.91 | 1050.05 | 372.97 | 367.62 | 292.33 | 271.09 | 311.74 | 400.99 |
| Italy | 337.11 | 339.66 | 338.63 | 321.70 | 316.60 | 322.78 | 325.20 | 327.90 | 329.32 | 340.40 |
| Japan | 185.13 | 192.16 | 201.74 | 212.48 | 224.91 | 242.92 | 273.56 | 301.71 | 318.03 | 334.86 |
| Kuwait | 86.12 | 29.85 | 68.14 | 48.95 | 52.86 | 61.73 | 58.66 | 67.57 | 73.94 | 58.82 |
| Latvia | 54.91 | 54.33 | 56.20 | 63.12 | 66.00 | 82.96 | 96.71 | 107.21 | 117.30 | 130.08 |
| Lithuania | 143.30 | 153.93 | 186.33 | 200.25 | 235.48 | 267.29 | 264.43 | 331.47 | 341.06 | 407.34 |
| Luxembourg | 574.27 | 557.09 | 509.76 | 483.4 | 359.87 | 336.84 | 348.33 | 340.29 | 372.83 | 406.92 |
| Netherlands | 1680.58 | 1656.53 | 1650.71 | 1625.65 | 1530.01 | 1459.85 | 1487.41 | 1413.58 | 1028.56 | 1181.41 |
| New Zealand | 849.56 | 854.3 | 877.53 | 875.69 | 925.94 | 967.01 | 982.14 | 1003.72 | 1011.49 | 1037.80 |
| Norway | 1084.64 | 1101.36 | 1159.37 | 1177.04 | 1232.47 | 1314.52 | 1392.91 | 1418.78 | 1551.18 | 1644.75 |
| Poland | 341.72 | 348.05 | 370.10 | 375.93 | 414.76 | 365.92 | 425.35 | 433.59 | 483.21 | 519.90 |
| Portugal | 101.77 | 105.09 | 120.30 | 123.65 | 145.82 | 145.66 | 157.23 | 159.14 | 188.33 | 194.39 |
| Romania | 27.54 | 35.03 | 32.29 | 37.73 | 43.56 | 45.20 | 50.26 | 56.19 | 58.82 | 59.19 |
| Russian Federation | 63.60 | 75.96 | 94.71 | 106.35 | 119.14 | 118.78 | 146.49 | 173.4 | 216.56 | 272.88 |
| Saudi Arabia | 1010.46 | 831.93 | 577.57 | 711.11 | 769.98 | 667.02 | 659.00 | 613.26 | 542.64 | 492.41 |
| Singapore | 25.12 | 28.27 | 30.20 | 31.80 | 34.78 | 36.64 | 48.66 | 47.21 | 45.65 | 54.91 |
| Slovak Republic | 205.89 | 216.83 | 219.63 | 224.28 | 240.92 | 257.33 | 268.68 | 282.52 | 296.58 | 298.51 |
| Slovenia | 268.46 | 257.05 | 256.08 | 256.70 | 256.07 | 271.94 | 273.31 | 284.67 | 288.97 | 288.36 |
| South Korea | 56.11 | 68.59 | 74.00 | 85.14 | 95.33 | 108.64 | 131.56 | 153.28 | 171.57 | 182.60 |
| Spain | 395.82 | 411.84 | 433.06 | 450.13 | 470.35 | 505.44 | 546.56 | 566.20 | 580.38 | 639.79 |
| Sweden | 682.32 | 705.94 | 732.94 | 766.61 | 806.54 | 824.28 | 863.31 | 873.97 | 903.49 | 960.08 |
| Switzerland | 536.99 | 543.45 | 539.85 | 548.03 | 544.58 | 569.79 | 577.25 | 589.86 | 595.24 | 608.59 |
| United Arab Emirates | 486.13 | 151.73 | 170.93 | 172.37 | 92.65 | 97.06 | 92.78 | 109.56 | 117.55 | 130.81 |
| United Kingdom | 2016.41 | 2020.41 | 1951.7 | 1888.21 | 1919.95 | 1651.55 | 1726.35 | 1771.03 | 1780.86 | 1859.33 |
| United States | 537.96 | 549.73 | 564.59 | 583.57 | 594.04 | 650.28 | 750.32 | 858.15 | 934.59 | 1045.2 |
| Uruguay | 4.56 | 4.15 | 6.66 | 6.03 | 8.23 | 9.47 | 9.57 | 9.69 | 12.66 | 12.34 |
| **Upper-middle-income countries/regions** | | | | | | | | | | |
| Algeria | 444.31 | 472.25 | 486.49 | 809.5 | 499.08 | 472.66 | 440.56 | 451.71 | 475.99 | 495.72 |
| Argentina | 11.21 | 11.06 | 11.61 | 12.11 | 12.11 | 13.00 | 14.45 | 14.89 | 15.34 | 16.49 |
| Belarus | 46.15 | 44.51 | 52.42 | 62.28 | 80.34 | 108.69 | 124.73 | 124.51 | 132.2 | 180.01 |
| Bosnia and Herzegovina | 2.41 | 2.24 | 5.09 | 10.77 | 17.97 | 21.68 | 31.65 | 43.57 | 51.00 | 59.87 |
| Brazil | 406.84 | 419.83 | 424.55 | 461.86 | 521.88 | 596.86 | 582.49 | 634.88 | 692.15 | 764.49 |
| China | 0.75 | 0.86 | 0.97 | 1.05 | 1.18 | 1.11 | 1.53 | 1.91 | 2.74 | 5.64 |
| Colombia | 9.47 | 9.02 | 8.59 | 8.79 | 8.28 | 8.26 | 8.74 | 9.08 | 9.60 | 10.65 |
| Dominican Republic | 0.29 | 0.53 | 0.47 | 0.68 | 0.63 | 0.58 | 0.49 | 0.49 | 0.42 | 0.69 |
| Ecuador | 1.14 | 3.26 | 4.10 | 4.16 | 3.37 | 3.59 | 3.28 | 3.59 | 4.13 | 3.91 |
| Indonesia | 8.94 | 6.52 | 1.97 | 3.08 | 2.02 | 1.73 | 1.82 | 2.16 | 0.44 | 0.39 |
| Kazakhstan | 9.13 | 8.77 | 9.48 | 10.71 | 12.08 | 16.83 | 21.66 | 25.29 | 32.23 | 40.85 |
| Malaysia | 22.75 | 14.32 | 10.22 | 8.49 | 6.87 | 5.68 | 6.74 | 10.09 | 10.84 | 9.59 |
| Mexico | 5.91 | 5.80 | 6.29 | 6.08 | 5.90 | 5.76 | 6.51 | 6.16 | 6.32 | 6.99 |
| Paraguay | 2.19 | 2.26 | 3.13 | 5.71 | 7.52 | 7.38 | 8.47 | 11.28 | 14.53 | 13.71 |
| Peru | 0.18 | 0.33 | 0.39 | 0.64 | 0.56 | 0.76 | 1.02 | 1.21 | 2.05 | 3.30 |
| Serbia | 31.67 | 41.97 | 45.92 | 56.64 | 60.76 | 67.20 | 76.03 | 94.71 | 116.53 | 129.73 |
| South Africa | 194.58 | 201.33 | 202.25 | 221.14 | 234.19 | 238.44 | 223.81 | 234.71 | 202.81 | 199.87 |
| Thailand | 61.86 | 46.40 | 56.16 | 103.19 | 55.49 | 45.21 | 58.13 | 50.91 | 55.95 | 51.39 |
| Turkiye | 90.00 | 90.30 | 92.36 | 86.96 | 82.06 | 76.95 | 90.74 | 88.05 | 101.37 | 115.79 |
| Venezuela | 9.34 | 8.48 | 7.14 | 3.73 | 2.96 | 6.32 | 5.35 | 4.68 | 4.13 | 6.03 |
| **Lower-middle-income countries/regions** | | | | | | | | | | |
| Bangladesh | 193.94 | 236.47 | 229.33 | 216.05 | 203.49 | 204.5 | 216.43 | 238.15 | 217.95 | 208.36 |
| Bolivia | 25.14 | 26.95 | 25.74 | 25.92 | 16.13 | 32.33 | 25.65 | 25.91 | 32.31 | 29.56 |
| Egypt | 7.87 | 10.21 | 10.29 | 12.8 | 13.82 | 15.48 | 24.54 | 33.26 | 29.73 | 32.88 |
| India | 3.37 | 3.20 | 3.28 | 3.84 | 3.80 | 3.35 | 3.69 | 3.89 | 4.08 | 4.26 |
| Jordan | 2.40 | 7.97 | 6.95 | 5.51 | 14.93 | 15.81 | 21.03 | 23.05 | 26.01 | 26.47 |
| Lebanon | 12.40 | 18.55 | 16.82 | 30.69 | 44.13 | 46.37 | 38.13 | 13.71 | 29.28 | 23.94 |
| Morocco | 100.90 | 115.3 | 111.04 | 3.53 | 3.80 | 4.34 | 6.83 | 5.73 | 6.15 | 8.38 |
| Pakistan | 241.00 | 218.83 | 194.36 | 215.26 | 232.66 | 218.38 | 86.07 | 260.27 | 302.97 | 269.42 |
| Philippines | 7.51 | 8.23 | 6.49 | 8.52 | 5.85 | 5.03 | 7.98 | 8.00 | 7.42 | 8.36 |
| Sri Lanka | 50.03 | 48.36 | 55.07 | 55.3 | 61.24 | 77.06 | 81.45 | 74.59 | 76.88 | 80.58 |
| Tunisia | 932.47 | 828.26 | 740.84 | 856.76 | 874.16 | 1052.22 | 877.72 | 811.99 | 803.09 | 705.48 |
| Vietnam | 0.22 | 0.57 | 0.67 | 0.85 | 3.25 | 2.07 | 1.15 | 1.17 | 0.47 | 0.10 |

**b) Acute migraine-specific medications**

|  | Consumption (standard units per 1,000 population) | | | | | | | | | |
| --- | --- | --- | --- | --- | --- | --- | --- | --- | --- | --- |
| Country/Region | 2015 | 2016 | 2017 | 2018 | 2019 | 2020 | 2021 | 2022 | 2023 | 2024 |
| **High-income countries/regions** | | | | | | | | | | |
| Australia | 224.44 | 241.53 | 252.21 | 280.17 | 299.31 | 309.71 | 334.14 | 340.15 | 348.19 | 367.27 |
| Austria | 240.27 | 246.05 | 247.90 | 255.09 | 252.70 | 250.29 | 264.26 | 273.70 | 272.19 | 284.26 |
| Belgium | 334.47 | 372.18 | 378.27 | 393.01 | 423.20 | 409.56 | 444.77 | 434.41 | 469.36 | 482.43 |
| Bulgaria | 27.10 | 30.11 | 35.24 | 39.86 | 42.33 | 41.96 | 45.64 | 51.93 | 60.13 | 63.74 |
| Canada | 433.20 | 442.50 | 437.01 | 428.53 | 443.76 | 457.26 | 464.52 | 466.57 | 473.64 | 485.63 |
| Chile | 136.19 | 145.42 | 151.03 | 157.18 | 167.94 | 184.31 | 230.33 | 229.40 | 227.45 | 244.73 |
| Croatia | 95.91 | 100.06 | 104.14 | 113.85 | 118.70 | 125.77 | 133.32 | 141.30 | 160.18 | 169.75 |
| Czech Republic | 247.22 | 256.70 | 279.82 | 283.25 | 297.91 | 300.96 | 308.54 | 324.08 | 308.35 | 301.87 |
| Estonia | 139.76 | 153.30 | 161.27 | 193.38 | 207.17 | 239.99 | 234.29 | 332.26 | 334.34 | 375.19 |
| Finland | 585.69 | 607.93 | 620.87 | 687.16 | 698.92 | 690.53 | 730.10 | 706.58 | 780.37 | 812.79 |
| France | 604.53 | 609.25 | 605.01 | 605.34 | 614.99 | 625.06 | 637.97 | 631.98 | 631.37 | 588.31 |
| Germany | 339.06 | 332.58 | 338.12 | 339.34 | 354.04 | 357.82 | 370.29 | 375.32 | 387.46 | 395.47 |
| Greece | 186.28 | 197.95 | 210.02 | 231.35 | 244.21 | 255.34 | 290.13 | 300.65 | 328.30 | 359.61 |
| Hong Kong, China | 19.83 | 14.86 | 14.81 | 18.26 | 15.24 | 28.06 | 30.17 | 34.42 | 40.53 | 37.82 |
| Hungary | 85.59 | 89.99 | 94.14 | 101.83 | 106.79 | 113.29 | 123.95 | 131.66 | 135.92 | 135.84 |
| Ireland | 181.47 | 184.95 | 196.57 | 207.38 | 224.95 | 249.56 | 265.30 | 260.76 | 286.06 | 327.46 |
| Italy | 301.08 | 304.38 | 304.78 | 311.33 | 316.23 | 321.84 | 323.55 | 325.47 | 326.23 | 335.27 |
| Japan | 168.21 | 174.87 | 184.79 | 196.27 | 208.50 | 226.12 | 255.72 | 281.87 | 296.60 | 312.72 |
| Kuwait | 54.84 | 29.85 | 56.10 | 35.07 | 45.46 | 54.05 | 51.00 | 66.01 | 72.03 | 42.11 |
| Latvia | 54.91 | 54.33 | 56.20 | 63.12 | 66.00 | 82.96 | 96.69 | 107.15 | 117.08 | 129.26 |
| Lithuania | 143.30 | 153.93 | 186.33 | 200.25 | 235.31 | 266.43 | 260.70 | 324.62 | 330.53 | 391.63 |
| Luxembourg | 316.20 | 322.04 | 322.11 | 325.11 | 330.94 | 329.66 | 338.00 | 327.29 | 357.35 | 385.21 |
| Netherlands | 534.15 | 531.46 | 535.65 | 542.29 | 534.33 | 510.65 | 535.38 | 563.18 | 567.78 | 580.11 |
| New Zealand | 547.65 | 570.81 | 614.02 | 640.57 | 675.24 | 722.69 | 745.21 | 768.87 | 794.12 | 824.10 |
| Norway | 1075.02 | 1092.13 | 1151.82 | 1170.43 | 1225.23 | 1297.67 | 1371.70 | 1394.62 | 1522.84 | 1546.99 |
| Poland | 92.76 | 101.13 | 112.91 | 119.72 | 138.73 | 144.54 | 162.30 | 174.41 | 223.92 | 265.13 |
| Portugal | 101.77 | 105.09 | 120.30 | 123.65 | 145.82 | 145.64 | 157.14 | 158.63 | 187.34 | 192.72 |
| Romania | 27.54 | 35.03 | 32.29 | 37.73 | 43.56 | 45.20 | 50.26 | 56.17 | 58.17 | 57.61 |
| Russian Federation | 63.60 | 75.96 | 94.71 | 106.35 | 119.14 | 118.78 | 146.45 | 173.29 | 216.44 | 272.71 |
| Saudi Arabia | 25.73 | 26.80 | 37.27 | 46.93 | 52.25 | 45.68 | 55.32 | 65.65 | 83.05 | 92.63 |
| Singapore | 25.12 | 28.27 | 30.20 | 31.78 | 34.59 | 36.36 | 48.31 | 46.86 | 45.23 | 54.38 |
| Slovak Republic | 175.22 | 185.13 | 189.64 | 193.57 | 204.70 | 213.90 | 219.59 | 225.89 | 231.57 | 228.43 |
| Slovenia | 268.46 | 257.05 | 256.08 | 256.69 | 256.06 | 270.41 | 270.46 | 280.32 | 283.03 | 280.00 |
| South Korea | 52.65 | 59.33 | 64.46 | 75.17 | 82.44 | 94.49 | 113.39 | 130.15 | 141.76 | 148.58 |
| Spain | 295.40 | 323.62 | 344.85 | 363.98 | 391.73 | 418.54 | 456.61 | 474.50 | 495.69 | 538.88 |
| Sweden | 682.32 | 705.94 | 732.94 | 766.58 | 804.11 | 820.55 | 858.44 | 867.88 | 896.34 | 935.07 |
| Switzerland | 536.99 | 543.45 | 539.85 | 547.83 | 542.42 | 566.64 | 572.81 | 584.23 | 588.47 | 597.50 |
| United Arab Emirates | 78.27 | 72.72 | 79.77 | 84.68 | 91.40 | 94.78 | 89.63 | 105.99 | 113.08 | 119.44 |
| United Kingdom | 400.77 | 449.30 | 458.34 | 460.24 | 499.65 | 519.33 | 537.05 | 560.49 | 595.05 | 641.74 |
| United States | 463.01 | 474.27 | 486.14 | 504.10 | 507.05 | 554.27 | 636.02 | 679.35 | 688.50 | 723.89 |
| Uruguay | 4.56 | 4.15 | 6.66 | 6.03 | 8.23 | 9.47 | 9.57 | 9.69 | 12.66 | 12.34 |
| **Upper-middle-income countries/regions** | | | | | | | | | | |
| Algeria | 5.51 | 7.06 | 7.30 | 7.12 | 2.98 | 15.36 | 15.46 | 15.85 | 20.06 | 29.12 |
| Argentina | 11.21 | 11.05 | 11.60 | 12.11 | 12.11 | 13.00 | 14.45 | 14.88 | 15.33 | 16.48 |
| Belarus | 46.15 | 44.51 | 52.42 | 62.28 | 80.34 | 108.69 | 124.73 | 124.51 | 132.20 | 180.01 |
| Bosnia and Herzegovina | 2.41 | 2.24 | 5.09 | 10.77 | 17.97 | 21.68 | 31.65 | 43.57 | 51.00 | 59.87 |
| Brazil | 406.81 | 419.82 | 424.55 | 461.86 | 521.87 | 596.77 | 582.29 | 634.68 | 691.93 | 764.18 |
| China | 0.65 | 0.74 | 0.82 | 0.89 | 0.90 | 0.80 | 0.97 | 1.22 | 1.87 | 3.73 |
| Colombia | 9.47 | 9.02 | 8.59 | 8.79 | 8.28 | 8.26 | 8.74 | 9.07 | 9.60 | 10.63 |
| Dominican Republic | 0.29 | 0.53 | 0.47 | 0.68 | 0.63 | 0.58 | 0.49 | 0.49 | 0.42 | 0.69 |
| Ecuador | 1.14 | 3.26 | 4.10 | 4.16 | 3.37 | 3.59 | 3.28 | 3.59 | 4.13 | 3.91 |
| Indonesia | 4.87 | 4.20 | 1.90 | 3.06 | 2.02 | 1.73 | 1.82 | 2.16 | 0.44 | 0.39 |
| Kazakhstan | 9.13 | 8.77 | 9.48 | 10.71 | 12.08 | 16.83 | 21.66 | 25.25 | 32.20 | 40.81 |
| Malaysia | 3.44 | 3.12 | 3.96 | 4.18 | 4.85 | 3.86 | 4.91 | 8.56 | 9.46 | 7.02 |
| Mexico | 5.90 | 5.80 | 6.29 | 6.08 | 5.90 | 5.75 | 6.49 | 6.11 | 6.20 | 6.88 |
| Paraguay | 2.19 | 2.26 | 3.13 | 5.71 | 7.52 | 7.38 | 8.47 | 11.28 | 14.53 | 13.71 |
| Peru | 0.18 | 0.33 | 0.39 | 0.64 | 0.56 | 0.76 | 1.02 | 1.21 | 2.05 | 3.30 |
| Serbia | 31.67 | 41.97 | 45.92 | 56.64 | 60.76 | 67.19 | 76.00 | 94.67 | 116.46 | 129.58 |
| South Africa | 11.09 | 10.86 | 11.32 | 11.31 | 11.63 | 10.22 | 10.85 | 11.38 | 13.20 | 14.01 |
| Thailand | 12.07 | 6.74 | 6.52 | 6.81 | 7.55 | 8.35 | 9.85 | 11.48 | 18.83 | 22.20 |
| Turkiye | 69.07 | 70.30 | 74.75 | 71.38 | 69.48 | 64.88 | 76.53 | 87.80 | 101.22 | 115.63 |
| Venezuela | 9.34 | 8.48 | 7.14 | 3.73 | 2.96 | 6.32 | 5.35 | 4.68 | 4.13 | 6.03 |
| **Lower-middle-income countries/regions** | | | | | | | | | | |
| Bangladesh | 7.22 | 9.56 | 11.76 | 11.46 | 13.38 | 15.70 | 29.89 | 38.33 | 39.64 | 33.23 |
| Bolivia | 25.14 | 26.95 | 25.74 | 25.92 | 16.13 | 32.33 | 25.65 | 25.91 | 32.31 | 29.56 |
| Egypt | 7.87 | 10.21 | 10.28 | 12.80 | 13.82 | 15.47 | 24.51 | 33.23 | 29.71 | 32.86 |
| India | 3.37 | 3.20 | 3.28 | 3.84 | 3.80 | 3.35 | 3.69 | 3.89 | 4.08 | 4.26 |
| Jordan | 2.40 | 7.97 | 6.95 | 5.51 | 14.93 | 15.81 | 21.01 | 23.02 | 25.98 | 26.44 |
| Lebanon | 12.40 | 18.55 | 16.82 | 30.69 | 38.05 | 44.24 | 36.01 | 12.23 | 17.11 | 23.93 |
| Morocco | 1.94 | 2.02 | 2.63 | 3.09 | 3.19 | 4.33 | 3.79 | 5.32 | 5.83 | 8.38 |
| Pakistan | 3.36 | 3.96 | 5.47 | 6.39 | 6.71 | 6.82 | 8.27 | 7.75 | 8.70 | 8.99 |
| Philippines | 0.89 | 0.92 | 0.89 | 0.82 | 0.88 | 0.73 | 0.75 | 0.93 | 0.76 | 0.53 |
| Sri Lanka | 0.22 | 0.95 | 7.85 | 10.28 | 16.22 | 23.60 | 35.88 | 32.76 | 37.95 | 41.57 |
| Tunisia | 2.90 | 2.96 | 2.54 | 2.92 | 3.09 | 3.74 | 5.38 | 6.60 | 6.04 | 6.16 |
| Vietnam | 0.22 | 0.57 | 0.67 | 0.85 | 3.25 | 2.07 | 1.15 | 1.17 | 0.47 | 0.10 |

**c) Preventive migraine-specific medications**

|  | Consumption (standard units per 1,000 population) | | | | | | | | | |
| --- | --- | --- | --- | --- | --- | --- | --- | --- | --- | --- |
| Country/Region | 2015 | 2016 | 2017 | 2018 | 2019 | 2020 | 2021 | 2022 | 2023 | 2024 |
| **High-income countries/regions** | | | | | | | | | | |
| Australia | 557.87 | 585.59 | 549.54 | 573.25 | 579.63 | 597.22 | 602.43 | 596.00 | 576.43 | 585.23 |
| Austria | 0 | 0 | 0 | 0.08 | 1.36 | 3.36 | 6.02 | 8.49 | 11.64 | 15.04 |
| Belgium | 430.08 | 404.38 | 391.03 | 381.12 | 51.8 | 0.04 | 2.59 | 6.35 | 8.31 | 14.62 |
| Bulgaria | 0 | 0 | 0 | 0 | 0 | 0.02 | 0.03 | 0.01 | 0.01 | 0 |
| Canada | 25.55 | 25.2 | 22.97 | 23.25 | 23.81 | 25.7 | 25.36 | 51.77 | 183.12 | 281.3 |
| Chile | 0 | 0 | 0 | 0 | 0.02 | 0.06 | 0.11 | 0.14 | 0.15 | 0.18 |
| Croatia | 0 | 0 | 0 | 0 | 0.77 | 2.07 | 3.68 | 5.42 | 7.85 | 10.88 |
| Czech Republic | 12.17 | 0 | 0 | 0 | 0.09 | 0.91 | 2.53 | 3.88 | 5.46 | 7.89 |
| Estonia | 0 | 0 | 0 | 0 | 0.07 | 0.12 | 0.13 | 0.28 | 0.41 | 2.94 |
| Finland | 0 | 0 | 0 | 0.21 | 5.26 | 8.01 | 9.53 | 11.33 | 12.97 | 34.34 |
| France | 313.10 | 319.87 | 280.16 | 265.07 | 268.69 | 236.52 | 262.69 | 256.95 | 243.75 | 167.32 |
| Germany | 18.45 | 16.52 | 16.17 | 16.36 | 17.77 | 17.96 | 17.02 | 16.89 | 20.17 | 23.15 |
| Greece | 0 | 0 | 0 | 0 | 0 | 0.03 | 0.05 | 0.07 | 0.10 | 0.15 |
| Hong Kong, China | 41.14 | 37.34 | 43.00 | 39.90 | 40.75 | 38.25 | 41.17 | 38.30 | 35.97 | 37.14 |
| Hungary | 402.01 | 391.04 | 374.97 | 379.39 | 327.94 | 299.12 | 327.14 | 328.83 | 292.51 | 269.12 |
| Ireland | 1614.15 | 2582.97 | 2745.34 | 842.66 | 148.02 | 118.07 | 27.03 | 10.32 | 25.68 | 73.53 |
| Italy | 36.03 | 35.29 | 33.84 | 10.37 | 0.38 | 0.95 | 1.65 | 2.43 | 3.09 | 5.14 |
| Japan | 16.92 | 17.29 | 16.96 | 16.21 | 16.40 | 16.79 | 17.84 | 19.84 | 21.42 | 22.14 |
| Kuwait | 3.46 | 9.26 | 9.54 | 9.97 | 12.89 | 14.15 | 18.17 | 23.13 | 29.81 | 34.03 |
| Latvia | 31.29 | 0 | 12.04 | 13.88 | 7.39 | 7.68 | 7.66 | 1.55 | 1.91 | 16.71 |
| Lithuania | 0 | 0 | 0 | 0 | 0 | 0 | 0.02 | 0.06 | 0.23 | 0.81 |
| Luxembourg | 0 | 0 | 0 | 0 | 0.17 | 0.85 | 3.73 | 6.85 | 10.53 | 15.71 |
| Netherlands | 258.07 | 235.05 | 187.65 | 158.29 | 28.93 | 7.17 | 10.33 | 13.01 | 15.48 | 21.71 |
| New Zealand | 1146.44 | 1125.07 | 1115.06 | 1083.36 | 995.68 | 949.20 | 952.02 | 850.40 | 460.78 | 601.30 |
| Norway | 301.92 | 283.49 | 263.50 | 235.11 | 250.69 | 244.32 | 236.93 | 234.85 | 217.37 | 213.70 |
| Poland | 9.62 | 9.22 | 7.55 | 6.61 | 7.24 | 16.85 | 21.21 | 24.16 | 28.33 | 97.76 |
| Portugal | 248.96 | 246.91 | 257.19 | 256.21 | 276.03 | 221.38 | 263.05 | 259.18 | 259.29 | 254.77 |
| Romania | 0 | 0 | 0 | 0 | 0 | 0.02 | 0.09 | 0.51 | 0.98 | 1.67 |
| Russian Federation | 0 | 0 | 0 | 0 | 0 | 0 | 0 | 0.02 | 0.66 | 1.58 |
| Saudi Arabia | 0 | 0 | 0 | 0 | 0 | 0 | 0.05 | 0.10 | 0.12 | 0.17 |
| Singapore | 984.73 | 805.13 | 540.30 | 664.18 | 717.73 | 621.33 | 603.68 | 547.61 | 459.59 | 399.78 |
| Slovak Republic | 0 | 0 | 0 | 0.02 | 0.18 | 0.27 | 0.35 | 0.36 | 0.42 | 0.53 |
| Slovenia | 30.66 | 31.70 | 29.99 | 30.71 | 36.22 | 43.44 | 49.09 | 56.63 | 65.01 | 70.08 |
| South Korea | 0 | 0 | 0 | 0 | 0.01 | 1.53 | 2.86 | 4.36 | 5.94 | 8.36 |
| Spain | 100.42 | 88.22 | 88.21 | 86.16 | 78.62 | 86.91 | 89.96 | 91.69 | 84.69 | 100.91 |
| Sweden | 0 | 0 | 0 | 0.02 | 2.43 | 3.73 | 4.87 | 6.09 | 7.16 | 25.01 |
| Switzerland | 0 | 0 | 0 | 0.20 | 2.16 | 3.14 | 4.45 | 5.62 | 6.77 | 11.09 |
| United Arab Emirates | 407.87 | 79.01 | 91.15 | 87.69 | 1.25 | 2.27 | 3.15 | 3.57 | 4.47 | 11.37 |
| United Kingdom | 1615.64 | 1571.12 | 1493.36 | 1427.97 | 1420.3 | 1132.21 | 1189.3 | 1210.54 | 1185.81 | 1217.58 |
| United States | 74.95 | 75.46 | 78.44 | 79.47 | 86.99 | 96.01 | 114.30 | 178.80 | 246.09 | 321.31 |
| Uruguay | 557.87 | 585.59 | 549.54 | 573.25 | 579.63 | 597.22 | 602.43 | 596.00 | 576.43 | 585.23 |
| **Upper-middle-income countries/regions** | | | | | | | | | | |
| Algeria | 438.80 | 465.20 | 479.20 | 802.38 | 496.10 | 457.30 | 425.10 | 435.86 | 455.93 | 466.60 |
| Argentina | 0 | 0.01 | 0 | 0 | 0 | 0 | 0 | 0.01 | 0.01 | 0.01 |
| Belarus | 0 | 0 | 0 | 0 | 0 | 0 | 0 | 0 | 0 | 0 |
| Bosnia and Herzegovina | 0 | 0 | 0 | 0 | 0 | 0 | 0 | 0 | 0 | 0 |
| Brazil | 0.03 | 0.01 | 0.01 | 0 | 0 | 0.09 | 0.20 | 0.20 | 0.22 | 0.31 |
| China | 0.10 | 0.12 | 0.15 | 0.17 | 0.28 | 0.32 | 0.56 | 0.69 | 0.88 | 1.92 |
| Colombia | 0 | 0 | 0 | 0 | 0 | 0 | 0 | 0.01 | 0.01 | 0.01 |
| Dominican Republic | 0 | 0 | 0 | 0 | 0 | 0 | 0 | 0 | 0 | 0 |
| Ecuador | 0 | 0 | 0 | 0 | 0 | 0 | 0 | 0 | 0 | 0 |
| Indonesia | 4.07 | 2.33 | 0.07 | 0.02 | 0 | 0 | 0 | 0 | 0 | 0 |
| Kazakhstan | 0 | 0 | 0 | 0 | 0 | 0 | 0.01 | 0.04 | 0.03 | 0.03 |
| Malaysia | 19.31 | 11.20 | 6.26 | 4.30 | 2.02 | 1.82 | 1.82 | 1.53 | 1.38 | 2.56 |
| Mexico | 0.01 | 0 | 0 | 0 | 0 | 0.01 | 0.02 | 0.05 | 0.11 | 0.11 |
| Paraguay | 0 | 0 | 0 | 0 | 0 | 0 | 0 | 0 | 0 | 0 |
| Peru | 0 | 0 | 0 | 0 | 0 | 0 | 0 | 0 | 0 | 0 |
| Serbia | 0 | 0 | 0 | 0 | 0 | 0 | 0.03 | 0.05 | 0.07 | 0.15 |
| South Africa | 183.49 | 190.48 | 190.92 | 209.82 | 222.56 | 228.22 | 212.96 | 223.32 | 189.61 | 185.86 |
| Thailand | 49.79 | 39.66 | 49.64 | 96.38 | 47.94 | 36.85 | 48.28 | 39.43 | 37.12 | 29.19 |
| Turkiye | 20.93 | 20.00 | 17.61 | 15.58 | 12.58 | 12.07 | 14.22 | 0.25 | 0.15 | 0.16 |
| Venezuela | 0 | 0 | 0 | 0 | 0 | 0 | 0 | 0 | 0 | 0 |
| **Lower-middle-income countries/regions** | | | | | | | | | | |
| Bangladesh | 186.71 | 226.91 | 217.57 | 204.59 | 190.12 | 188.80 | 186.54 | 199.82 | 178.30 | 175.13 |
| Bolivia | 0 | 0 | 0 | 0 | 0 | 0 | 0 | 0 | 0 | 0 |
| Egypt | 0.01 | 0 | 0.01 | 0 | 0 | 0.02 | 0.03 | 0.03 | 0.02 | 0.02 |
| India | 0 | 0 | 0 | 0 | 0 | 0 | 0 | 0 | 0 | 0 |
| Jordan | 0 | 0 | 0 | 0 | 0 | 0 | 0.02 | 0.03 | 0.03 | 0.03 |
| Lebanon | 0 | 0 | 0 | 0 | 6.09 | 2.13 | 2.12 | 1.48 | 12.16 | 0.01 |
| Morocco | 98.96 | 113.28 | 108.41 | 0.44 | 0.61 | 0 | 3.03 | 0.41 | 0.31 | 0 |
| Pakistan | 237.64 | 214.87 | 188.89 | 208.87 | 225.94 | 211.57 | 77.8 | 252.53 | 294.27 | 260.43 |
| Philippines | 6.61 | 7.31 | 5.59 | 7.71 | 4.97 | 4.30 | 7.23 | 7.06 | 6.66 | 7.83 |
| Sri Lanka | 49.82 | 47.41 | 47.21 | 45.02 | 45.03 | 53.46 | 45.57 | 41.83 | 38.93 | 39.01 |
| Tunisia | 929.58 | 825.3 | 738.29 | 853.84 | 871.07 | 1048.48 | 872.34 | 805.40 | 797.05 | 699.33 |
| Vietnam | 0 | 0 | 0 | 0 | 0 | 0 | 0 | 0 | 0 | 0 |

**d) Propranolol**

|  | Consumption (standard units per 1,000 population) | | | | | | | | | |
| --- | --- | --- | --- | --- | --- | --- | --- | --- | --- | --- |
| Country/Region | 2015 | 2016 | 2017 | 2018 | 2019 | 2020 | 2021 | 2022 | 2023 | 2024 |
| **High-income countries/regions** | | | | | | | | | | |
| Australia | 2497.07 | 2603.86 | 2626.62 | 2737.90 | 2908.81 | 3127.14 | 3257.19 | 3496.28 | 3496.64 | 3720.88 |
| Austria | 1298.87 | 1328.09 | 1352.42 | 1384.12 | 1406.56 | 1435.88 | 1445.02 | 1452.48 | 1535.06 | 1166.54 |
| Belgium | 3674.11 | 3612.90 | 3505.11 | 3467.43 | 3389.74 | 3508.96 | 3667.40 | 2932.92 | 3229.28 | 3115.86 |
| Bulgaria | 1148.31 | 1127.96 | 1110.66 | 1082.57 | 1093.87 | 1128.99 | 1128.85 | 1122.09 | 1167.23 | 1171.31 |
| Canada | 1689.54 | 1757.25 | 1781.95 | 2077.65 | 2179.78 | 2290.84 | 2332.42 | 2523.12 | 2621.18 | 2404.01 |
| Chile | 993.85 | 814.71 | 747.07 | 738.97 | 750.51 | 721.36 | 946.13 | 557.79 | 573.42 | 592.93 |
| Croatia | 144.74 | 545.41 | 591.27 | 646.49 | 714.75 | 758.35 | 794.95 | 672.84 | 658.69 | 178.51 |
| Czech Republic | 0.00 | 0.68 | 1.13 | 3.48 | 5.44 | 6.77 | 5.80 | 5.90 | 4.83 | 4.40 |
| Estonia | 745.78 | 773.91 | 785.27 | 823.65 | 866.61 | 902.43 | 905.49 | 1011.31 | 1093.24 | 1154.05 |
| Finland | 4552.36 | 4689.09 | 4984.45 | 5235.12 | 5588.92 | 5679.79 | 5904.64 | 6367.71 | 6862.67 | 7155.05 |
| France | 3185.24 | 3197.23 | 3180.30 | 3165.03 | 3201.78 | 3270.98 | 3269.96 | 3235.11 | 3219.81 | 3235.21 |
| Germany | 1265.05 | 1254.63 | 1225.14 | 1203.33 | 1228.54 | 1226.78 | 1218.40 | 1303.57 | 1276.20 | 1317.48 |
| Greece | 1276.31 | 1292.83 | 1314.06 | 1353.05 | 1441.56 | 1474.94 | 1517.96 | 1532.48 | 1558.78 | 1524.83 |
| Hong Kong, China | 4834.06 | 4246.70 | 4165.66 | 4610.47 | 4147.70 | 4696.02 | 4859.41 | 5042.79 | 4915.26 | 4954.36 |
| Hungary | 1179.91 | 1173.62 | 1163.58 | 1151.42 | 1158.70 | 1186.46 | 1134.29 | 1095.11 | 1150.79 | 1034.94 |
| Ireland | 2118.57 | 2220.34 | 2358.29 | 1286.19 | 1143.27 | 1550.50 | 1379.36 | 1417.36 | 1445.14 | 1753.87 |
| Italy | 933.23 | 942.36 | 949.03 | 958.50 | 975.84 | 990.50 | 1000.68 | 1010.21 | 1037.42 | 929.80 |
| Japan | 565.81 | 555.92 | 547.95 | 548.14 | 536.57 | 539.20 | 540.25 | 501.37 | 523.81 | 549.24 |
| Kuwait | 1067.13 | 951.04 | 974.09 | 852.67 | 247.94 | 661.52 | 970.84 | 534.95 | 999.87 | 567.00 |
| Latvia | 665.74 | 609.59 | 619.98 | 637.57 | 654.32 | 689.22 | 742.25 | 772.01 | 810.18 | 852.11 |
| Lithuania | 333.53 | 381.31 | 429.13 | 382.91 | 460.90 | 497.88 | 529.16 | 638.66 | 607.10 | 716.87 |
| Luxembourg | 1518.24 | 1501.67 | 1491.74 | 1477.97 | 1512.62 | 1557.39 | 1612.48 | 1055.62 | 1469.61 | 1629.99 |
| Netherlands | 1983.80 | 1946.51 | 1906.61 | 1849.60 | 1800.27 | 1727.02 | 1794.61 | 1854.61 | 1805.72 | 1865.74 |
| New Zealand | 1223.83 | 1221.04 | 1250.77 | 1357.02 | 1476.03 | 1579.78 | 1679.62 | 1860.77 | 2071.81 | 2209.38 |
| Norway | 1295.55 | 1365.18 | 1431.82 | 1286.96 | 1405.75 | 1347.02 | 1382.94 | 1563.07 | 1656.87 | 1749.17 |
| Poland | 2187.31 | 2305.63 | 2285.56 | 2302.92 | 2348.72 | 2456.16 | 2567.19 | 2679.77 | 2672.42 | 2834.64 |
| Portugal | 4604.29 | 4734.55 | 4908.81 | 5138.17 | 4915.06 | 5388.69 | 5628.53 | 4230.16 | 4295.35 | 4143.51 |
| Romania | 1022.93 | 994.01 | 1082.80 | 1152.91 | 1191.50 | 1169.16 | 1158.36 | 1214.97 | 1209.83 | 1264.94 |
| Russian Federation | 1814.16 | 1877.97 | 2147.28 | 2214.01 | 1021.81 | 1018.42 | 1317.87 | 1274.08 | 1383.27 | 1373.01 |
| Saudi Arabia | 597.46 | 576.75 | 790.63 | 717.33 | 1263.37 | 801.90 | 1025.82 | 1101.16 | 1155.82 | 1144.54 |
| Singapore | 1352.48 | 902.58 | 1013.32 | 762.95 | 778.11 | 771.39 | 1038.59 | 569.98 | 362.98 | 395.74 |
| Slovak Republic | 0.49 | 2.85 | 4.65 | 5.77 | 7.03 | 9.14 | 9.64 | 11.57 | 11.48 | 11.56 |
| Slovenia | 1097.37 | 1236.85 | 1273.01 | 1313.40 | 1347.55 | 1354.20 | 1356.37 | 1379.33 | 1098.01 | 540.02 |
| South Korea | 2819.94 | 2899.34 | 3118.47 | 3520.97 | 3839.31 | 3959.57 | 4281.82 | 4538.78 | 4615.41 | 4750.40 |
| Spain | 2273.43 | 2416.65 | 2458.37 | 2456.56 | 2500.16 | 2416.46 | 2595.28 | 2651.27 | 2655.31 | 2575.49 |
| Sweden | 2963.76 | 3022.20 | 3072.30 | 3128.64 | 3151.42 | 3219.68 | 3361.44 | 3450.48 | 3589.73 | 3080.67 |
| Switzerland | 1958.03 | 1928.31 | 1837.06 | 1753.25 | 1695.52 | 1730.93 | 1718.05 | 1727.97 | 1703.85 | 1761.31 |
| United Arab Emirates | 1256.79 | 1559.90 | 1186.30 | 1185.92 | 1038.34 | 696.44 | 628.32 | 605.96 | 746.51 | 831.05 |
| United Kingdom | 4714.14 | 5482.17 | 5783.76 | 6063.93 | 6578.40 | 7035.38 | 7405.45 | 7811.08 | 8433.40 | 8994.95 |
| United States | 2096.75 | 2087.04 | 2261.32 | 2377.71 | 2514.51 | 2735.23 | 2917.82 | 3089.56 | 3253.43 | 3582.87 |
| Uruguay | 289.33 | 280.12 | 275.04 | 264.84 | 267.64 | 307.83 | 309.58 | 300.22 | 285.21 | 264.99 |
| **Upper-middle-income countries/regions** | | | | | | | | | | |
| Algeria | 769.38 | 186.67 | 1.07 | 154.16 | 339.11 | 202.20 | 278.38 | 259.00 | 124.66 | 91.39 |
| Argentina | 389.35 | 388.36 | 377.63 | 361.64 | 349.01 | 362.45 | 384.38 | 362.96 | 368.51 | 347.01 |
| Belarus | 1587.45 | 1431.37 | 1457.25 | 1401.91 | 1604.00 | 1487.22 | 1324.45 | 1187.26 | 1028.44 | 1197.05 |
| Bosnia and Herzegovina | 1101.12 | 1156.48 | 1155.31 | 1166.02 | 1186.84 | 1241.34 | 1192.02 | 1194.83 | 1299.50 | 1341.88 |
| Brazil | 2167.72 | 2133.22 | 2162.12 | 2208.31 | 2316.48 | 2476.56 | 2331.69 | 2173.72 | 2122.60 | 2084.95 |
| China | 259.78 | 237.50 | 237.05 | 227.36 | 241.10 | 235.83 | 245.39 | 261.89 | 284.35 | 293.40 |
| Colombia | 182.06 | 159.87 | 164.26 | 191.64 | 163.94 | 163.46 | 165.70 | 157.44 | 142.47 | 163.89 |
| Dominican Republic | 262.62 | 273.58 | 271.65 | 257.14 | 243.24 | 212.34 | 182.39 | 171.98 | 154.53 | 176.52 |
| Ecuador | 190.67 | 197.29 | 226.32 | 239.69 | 275.13 | 303.24 | 329.32 | 359.52 | 340.42 | 345.05 |
| Indonesia | 107.30 | 97.83 | 146.38 | 144.07 | 174.87 | 161.41 | 156.94 | 174.70 | 184.37 | 207.14 |
| Kazakhstan | 858.78 | 918.84 | 851.93 | 830.62 | 353.51 | 33.67 | 103.22 | 148.24 | 123.16 | 5.62 |
| Malaysia | 609.50 | 562.44 | 603.41 | 679.40 | 589.98 | 578.49 | 602.38 | 710.45 | 622.40 | 574.03 |
| Mexico | 304.58 | 325.27 | 345.00 | 333.63 | 317.13 | 312.65 | 292.00 | 303.02 | 284.99 | 294.27 |
| Paraguay | 176.42 | 156.98 | 205.77 | 234.00 | 256.36 | 290.74 | 326.92 | 305.59 | 292.57 | 294.33 |
| Peru | 195.48 | 170.76 | 173.61 | 167.35 | 173.25 | 145.86 | 373.38 | 114.94 | 131.30 | 111.09 |
| Serbia | 2643.11 | 2936.64 | 3298.21 | 3141.46 | 3217.44 | 3534.30 | 3469.03 | 3474.95 | 3406.34 | 3822.51 |
| South Africa | 480.57 | 576.99 | 526.31 | 636.74 | 450.90 | 646.96 | 495.81 | 502.31 | 519.85 | 530.76 |
| Thailand | 2178.47 | 2104.14 | 2120.07 | 2695.05 | 2686.02 | 2994.03 | 2926.92 | 3032.75 | 3114.42 | 2912.71 |
| Turkiye | 1164.94 | 1238.16 | 1348.21 | 1436.93 | 1516.79 | 1569.64 | 1642.25 | 1920.15 | 1925.08 | 2019.95 |
| Venezuela | 337.79 | 320.70 | 312.07 | 241.19 | 108.54 | 155.19 | 150.96 | 179.71 | 189.65 | 246.86 |
| **Lower-middle-income countries/regions** | | | | | | | | | | |
| Bangladesh | 1505.59 | 2006.54 | 1873.01 | 1992.20 | 1988.45 | 2047.65 | 2032.58 | 1996.17 | 1631.75 | 1996.74 |
| Bolivia | 47.42 | 35.69 | 26.73 | 24.74 | 22.50 | 35.92 | 48.13 | 47.45 | 10.96 | 11.56 |
| Egypt | 751.79 | 923.04 | 578.06 | 767.23 | 978.77 | 843.90 | 903.21 | 928.38 | 1010.38 | 1017.90 |
| India | 354.04 | 366.50 | 384.14 | 411.10 | 423.32 | 433.26 | 453.79 | 443.57 | 438.09 | 432.43 |
| Jordan | 334.03 | 317.38 | 282.87 | 335.99 | 295.81 | 270.85 | 382.60 | 332.51 | 492.98 | 508.92 |
| Lebanon | 1538.36 | 1187.53 | 1097.56 | 1445.48 | 1592.21 | 1668.42 | 1778.18 | 1185.32 | 1243.13 | 1509.02 |
| Morocco | 441.35 | 446.88 | 450.14 | 434.72 | 480.64 | 511.21 | 568.22 | 556.26 | 576.56 | 592.73 |
| Pakistan | 1226.86 | 1131.48 | 1168.36 | 1317.23 | 1403.41 | 1256.09 | 1223.16 | 1314.18 | 1120.02 | 1167.94 |
| Philippines | 117.67 | 119.90 | 134.56 | 133.55 | 130.32 | 159.60 | 149.65 | 68.10 | 33.83 | 44.32 |
| Sri Lanka | 321.65 | 291.20 | 238.89 | 210.20 | 221.85 | 277.43 | 257.27 | 264.78 | 186.28 | 158.97 |
| Tunisia | 1077.64 | 1314.99 | 1349.85 | 1230.74 | 1051.11 | 1036.33 | 1023.57 | 1058.19 | 970.95 | 1194.75 |
| Vietnam | 148.90 | 155.97 | 141.43 | 138.41 | 62.94 | 38.48 | 42.97 | 41.13 | 45.23 | 67.02 |

Table S4. Consumption of acute migraine-specific medications by income-level group, 2015-2024

**a) Sumatriptan**

| Metrics | 2015 | 2016 | 2017 | 2018 | 2019 | 2020 | 2021 | 2022 | 2023 | 2024 |
| --- | --- | --- | --- | --- | --- | --- | --- | --- | --- | --- |
| **All regions** | | | | | | | | | | |
| Number of countries | 69 | 69 | 69 | 68 | 70 | 68 | 68 | 68 | 68 | 68 |
| Median (IQR^a^) | 23.07 (1.82–80.22) | 24.39 (1.88–85.59) | 25.54 (2.46–90.97) | 30.43 (2.50–99.38) | 33.08 (3.03–111.63) | 32.79 (3.23–113.67) | 36.87 (3.35–123.92) | 41.80 (3.83–138.52) | 41.20 (4.43–147.78) | 39.90 (5.26–154.33) |
| Unweighted mean (SE^b^) | 67.66 (12.44) | 69.67 (12.73) | 73.18 (13.25) | 76.95 (13.78) | 81.59 (14.34) | 84.83 (14.73) | 89.68 (15.44) | 95.34 (16.04) | 98.94 (16.58) | 101.39 (17.29) |
| Population-weighted mean (SE)^c^ | 32.02 (9.86) | 33.18 (10.19) | 34.40 (10.45) | 35.67 (10.77) | 36.65 (10.95) | 37.53 (11.21) | 40.83 (11.98) | 42.22 (12.15) | 43.19 (12.20) | 43.98 (12.46) |
| **High-income countries/regions** | | | | | | | | | | |
| Number of countries | 41 | 41 | 41 | 41 | 41 | 40 | 40 | 40 | 40 | 40 |
| Median (IQR) | 71.41 (27.34–149.51) | 77.31 (27.29–148.09) | 88.42 (30.75–157.18) | 92.79 (33.76–173.44) | 101.15 (37.51–200.94) | 109.42 (37.61–211.79) | 122.65 (40.90–209.18) | 124.40 (45.48–277.04) | 125.12 (46.66–275.83) | 128.48 (46.46–287.58) |
| Unweighted mean (SE) | 113.54 (18.51) | 116.79 (18.92) | 122.45 (19.66) | 128.43 (20.40) | 135.59 (21.14) | 140.15 (21.69) | 147.55 (22.73) | 156.51 (23.42) | 161.46 (24.23) | 164.60 (25.48) |
| Population-weighted mean (SE) | 136.33 (18.81) | 142.06 (19.30) | 148.07 (19.51) | 154.12 (19.95) | 158.97 (19.98) | 163.02 (20.50) | 176.55 (21.73) | 181.68 (21.63) | 184.75 (21.31) | 186.91 (22.02) |
| **Upper-middle-income countries/regions** | | | | | | | | | | |
| Number of countries | 17 | 17 | 17 | 17 | 18 | 18 | 18 | 18 | 18 | 18 |
| Median (IQR) | 2.30 (1.22–8.10) | 2.11 (1.14–7.79) | 3.00 (1.36–6.70) | 3.41 (1.52–5.19) | 3.18 (1.99–6.93) | 3.69 (1.72–10.63) | 4.58 (2.28–12.01) | 4.40 (2.54–14.34) | 6.10 (2.41–15.61) | 5.57 (2.44–18.34) |
| Unweighted mean (SE) | 7.59 (2.71) | 7.58 (2.78) | 8.36 (3.13) | 9.26 (3.60) | 10.78 (4.38) | 12.97 (5.28) | 14.73 (6.10) | 16.55 (6.69) | 19.63 (8.29) | 22.80 (9.98) |
| Population-weighted mean (SE) | 3.18 (2.31) | 3.01 (2.22) | 3.05 (2.24) | 3.16 (2.37) | 3.22 (2.47) | 3.62 (2.81) | 4.01 (3.07) | 4.42 (3.37) | 5.24 (3.98) | 6.46 (4.99) |
| **Lower-middle-income countries/regions** | | | | | | | | | | |
| Number of countries | 11 | 11 | 11 | 10 | 11 | 10 | 10 | 10 | 10 | 10 |
| Median (IQR) | 0.62 (0.21–1.94) | 0.77 (0.55–2.99) | 0.82 (0.47–2.59) | 0.94 (0.61–2.92) | 0.99 (0.59–3.13) | 1.38 (0.51–2.49) | 1.67 (0.76–5.60) | 1.82 (0.90–3.91) | 1.47 (0.66–3.46) | 1.18 (0.09–3.33) |
| Unweighted mean (SE) | 0.99 (0.29) | 1.78 (0.53) | 2.04 (0.72) | 2.57 (1.17) | 3.34 (1.47) | 3.59 (1.70) | 4.42 (1.64) | 4.52 (1.87) | 4.32 (1.92) | 3.31 (1.45) |
| Population-weighted mean (SE) | 0.68 (0.17) | 0.80 (0.27) | 0.83 (0.28) | 0.92 (0.36) | 0.97 (0.48) | 0.89 (0.50) | 1.99 (1.38) | 2.41 (1.81) | 2.26 (1.71) | 1.75 (1.07) |
| ^a^ IQR: Interquartile range;  ^b^ SE: Standard error;  ^c^ The population-weighted mean was calculated by assigning weights proportional to each country's population size for the respective year. The standard error of the weighted mean was derived from the unbiased weighted sample variance to account for the weights of different population sizes. | | | | | | | | | | |

**b) N02CA ergot alkaloids (ergotamine and dihydroergotamine)**

| Metrics | 2015 | 2016 | 2017 | 2018 | 2019 | 2020 | 2021 | 2022 | 2023 | 2024 |
| --- | --- | --- | --- | --- | --- | --- | --- | --- | --- | --- |
| **All regions** | | | | | | | | | | |
| Number of countries | 10 | 9 | 8 | 8 | 7 | 8 | 7 | 7 | 8 | 7 |
| Median (IQR^a^) | 4.35 (0.05–12.96) | 1.09 (0.04–13.50) | 0.74 (0.01–12.74) | 0.66 (0.04–13.20) | 0.56 (0.00–7.95) | 0.54 (0.03–15.72) | 0.46 (0.00–12.10) | 0.39 (0.00–11.97) | 0.38 (0.00–15.22) | 0.28 (0.00–12.69) |
| Unweighted mean (SE^b^) | 23.01 (15.72) | 22.83 (16.14) | 20.28 (14.23) | 20.76 (14.48) | 23.21 (18.07) | 27.45 (21.03) | 23.77 (17.98) | 27.01 (21.20) | 30.69 (24.62) | 31.30 (26.50) |
| Population-weighted mean (SE)^c^ | 15.97 (18.21) | 15.99 (18.64) | 13.83 (16.39) | 14.18 (16.66) | 17.33 (20.81) | 20.11 (24.28) | 17.21 (20.74) | 20.16 (24.44) | 23.05 (28.36) | 24.57 (30.44) |
| **High-income countries/regions** | | | | | | | | | | |
| Number of countries | 4 | 4 | 4 | 4 | 4 | 4 | 4 | 4 | 4 | 4 |
| Median (IQR) | 4.02 (0.64–15.03) | 3.88 (0.63–14.71) | 3.56 (0.53–13.89) | 3.23 (0.56–13.61) | 3.14 (0.48–13.18) | 2.62 (0.45–11.87) | 2.66 (0.39–12.25) | 2.41 (0.33–11.86) | 2.35 (0.30–11.19) | 1.94 (0.26–8.12) |
| Unweighted mean (SE) | 11.66 (9.12) | 11.46 (9.00) | 10.86 (8.61) | 10.94 (8.79) | 10.52 (8.45) | 9.70 (7.95) | 9.97 (8.21) | 9.78 (8.17) | 9.14 (7.62) | 6.44 (5.19) |
| Population-weighted mean (SE) | 1.60 (4.36) | 1.55 (4.27) | 1.44 (4.05) | 1.57 (4.05) | 1.48 (3.90) | 1.31 (3.63) | 1.31 (3.74) | 1.24 (3.70) | 1.11 (3.46) | 0.83 (2.39) |
| **Upper-middle-income countries/regions** | | | | | | | | | | |
| Number of countries | 4 | 4 | 3 | 3 | 2 | 3 | 2 | 2 | 3 | 2 |
| Median (IQR) | 4.35 (0.02–7.10) | 1.09 (0.07–3.80) | 0.74 (0.00–1.54) | 0.09 (0.00–2.77) | 0.00 (0.00–1.91) | 0.07 (0.00–1.67) | 0.00 (0.00–1.75) | 0.00 (0.00–2.08) | 0.00 (0.00–0.38) | 0.00 (0.00–0.28) |
| Unweighted mean (SE) | 37.54 (34.70) | 37.04 (35.81) | 32.11 (31.55) | 32.80 (32.09) | 40.60 (40.13) | 47.28 (46.85) | 40.45 (40.01) | 47.71 (47.19) | 54.97 (54.88) | 59.04 (58.97) |
| Population-weighted mean (SE) | 61.84 (44.25) | 62.18 (45.64) | 53.75 (40.40) | 55.05 (40.93) | 67.92 (51.33) | 78.87 (59.96) | 67.39 (51.14) | 79.29 (60.27) | 90.93 (70.28) | 97.42 (75.50) |
| **Lower-middle-income countries/regions** | | | | | | | | | | |
| Number of countries | 2 | 1 | 1 | 1 | 1 | 1 | 1 | 1 | 1 | 1 |
| Median (IQR) | 9.40 (4.74–14.07) | 10.04 (5.02–15.05) | 9.53 (4.76–14.29) | 10.30 (5.15–15.45) | 5.09 (2.54–7.63) | 13.37 (6.68–20.05) | 9.66 (4.83–14.49) | 9.75 (4.87–14.62) | 13.06 (6.53–19.60) | 11.68 (5.84–17.52) |
| Unweighted mean (SE) | 9.40 (9.33) | 10.04 (10.04) | 9.53 (9.53) | 10.30 (10.30) | 5.09 (5.09) | 13.37 (13.37) | 9.66 (9.66) | 9.75 (9.75) | 13.06 (13.06) | 11.68 (11.68) |
| Population-weighted mean (SE) | 0.22 (9.33) | 0.17 (10.04) | 0.16 (9.53) | 0.17 (10.30) | 0.08 (5.09) | 0.22 (13.37) | 0.16 (9.66) | 0.16 (9.75) | 0.22 (13.06) | 0.20 (11.68) |
| ^a^ IQR: Interquartile range;  ^b^ SE: Standard error;  ^c^ The population-weighted mean was calculated by assigning weights proportional to each country's population size for the respective year. The standard error of the weighted mean was derived from the unbiased weighted sample variance to account for the weights of different population sizes. | | | | | | | | | | |

**c) N02CC selective serotonin (5HT1) agonists (almotriptan, eletriptan, frovatriptan, lasmiditan, naratriptan, rizatriptan and zolmitriptan) except sumatriptan**

| Metrics | 2015 | 2016 | 2017 | 2018 | 2019 | 2020 | 2021 | 2022 | 2023 | 2024 |
| --- | --- | --- | --- | --- | --- | --- | --- | --- | --- | --- |
| **All regions** | | | | | | | | | | |
| Number of countries | 67 | 67 | 67 | 67 | 66 | 67 | 67 | 68 | 68 | 68 |
| Median (IQR^a^) | 10.92 (3.12–125.74) | 14.29 (3.45–138.79) | 13.96 (4.59–142.42) | 16.81 (5.29–154.98) | 20.85 (4.60–166.09) | 26.21 (4.78–178.32) | 31.75 (5.83–190.98) | 30.39 (6.62–187.77) | 40.98 (7.37–198.83) | 52.48 (10.09–214.71) |
| Unweighted mean (SE^b^) | 86.06 (15.88) | 89.40 (16.44) | 92.67 (16.73) | 95.95 (17.12) | 99.86 (17.66) | 104.70 (18.45) | 111.01 (19.28) | 112.95 (19.40) | 119.60 (20.46) | 126.18 (20.62) |
| Population-weighted mean (SE)^c^ | 47.95 (12.63) | 49.58 (12.96) | 51.65 (13.26) | 54.14 (13.72) | 56.48 (14.23) | 59.67 (15.04) | 63.38 (15.71) | 65.37 (15.95) | 67.67 (16.26) | 72.41 (16.77) |
| **High-income countries/regions** | | | | | | | | | | |
| Number of countries | 41 | 40 | 40 | 40 | 40 | 40 | 40 | 40 | 40 | 40 |
| Median (IQR) | 108.22 (17.50–201.01) | 107.64 (19.06–205.36) | 119.79 (21.84–207.47) | 122.81 (24.86–213.79) | 139.41 (23.79–206.37) | 145.00 (27.77–215.44) | 156.48 (33.76–230.84) | 151.34 (37.33–231.16) | 170.52 (45.25–230.53) | 174.64 (60.37–253.17) |
| Unweighted mean (SE) | 138.26 (23.50) | 143.22 (24.36) | 147.82 (24.61) | 152.07 (25.06) | 157.81 (25.79) | 164.35 (26.88) | 173.40 (28.07) | 175.93 (28.11) | 185.98 (29.68) | 194.18 (29.45) |
| Population-weighted mean (SE) | 159.32 (22.28) | 164.33 (22.67) | 168.48 (22.53) | 173.74 (22.56) | 180.77 (22.98) | 188.86 (23.63) | 200.59 (24.25) | 205.13 (24.06) | 210.38 (23.97) | 220.39 (22.57) |
| **Upper-middle-income countries/regions** | | | | | | | | | | |
| Number of countries | 16 | 17 | 17 | 17 | 17 | 17 | 17 | 18 | 18 | 18 |
| Median (IQR) | 3.33 (0.31–6.40) | 3.84 (0.37–7.70) | 3.92 (0.64–7.80) | 4.92 (1.18–8.90) | 4.46 (0.70–9.93) | 4.81 (1.52–9.37) | 5.16 (1.01–10.05) | 7.52 (1.75–10.86) | 6.20 (1.90–13.67) | 8.79 (3.38–18.77) |
| Unweighted mean (SE) | 17.68 (11.52) | 18.86 (12.05) | 20.82 (13.52) | 23.24 (15.22) | 24.26 (16.32) | 27.34 (18.36) | 29.87 (19.40) | 32.00 (20.14) | 34.24 (21.10) | 39.93 (23.34) |
| Population-weighted mean (SE) | 22.74 (18.32) | 23.93 (19.20) | 26.71 (21.59) | 29.82 (24.50) | 31.63 (26.29) | 35.31 (29.71) | 37.63 (31.30) | 39.58 (32.48) | 42.05 (33.80) | 47.99 (37.34) |
| **Lower-middle-income countries/regions** | | | | | | | | | | |
| Number of countries | 10 | 10 | 10 | 10 | 9 | 10 | 10 | 10 | 10 | 10 |
| Median (IQR) | 2.63 (1.02–5.12) | 2.52 (1.14–5.86) | 4.31 (2.00–7.64) | 3.40 (1.80–10.08) | 3.71 (2.69–13.14) | 4.33 (3.12–14.73) | 5.21 (3.29–19.06) | 5.55 (3.50–14.27) | 7.10 (3.41–18.70) | 8.38 (3.48–23.09) |
| Unweighted mean (SE) | 3.38 (0.97) | 4.21 (1.39) | 4.67 (1.14) | 5.74 (1.62) | 7.57 (2.28) | 8.95 (2.76) | 11.24 (3.59) | 10.67 (3.48) | 11.87 (3.78) | 13.86 (4.13) |
| Population-weighted mean (SE) | 3.00 (0.67) | 3.25 (0.95) | 3.70 (1.15) | 4.23 (1.21) | 4.64 (1.59) | 4.76 (1.95) | 5.67 (2.59) | 6.40 (3.18) | 6.75 (3.26) | 7.16 (3.45) |
| ^a^ IQR: Interquartile range;  ^b^ SE: Standard error;  ^c^ The population-weighted mean was calculated by assigning weights proportional to each country's population size for the respective year. The standard error of the weighted mean was derived from the unbiased weighted sample variance to account for the weights of different population sizes. | | | | | | | | | | |

**d) N02CD calcitonin gene-related peptide (CGRP) antagonists (rimegepant, ubrogepant and zavegepant)**

| Metrics | 2015 | 2016 | 2017 | 2018 | 2019 | 2020 | 2021 | 2022 | 2023 | 2024 |
| --- | --- | --- | --- | --- | --- | --- | --- | --- | --- | --- |
| **All regions** | | | | | | | | | | |
| Number of countries | 0 | 0 | 0 | 0 | 0 | 1 | 2 | 19 | 40 | 46 |
| Median (IQR^a^) | 0 | 0 | 0 | 0 | 0 | < 0.01 | < 0.01 | < 0.01 | 0.26 (0.05–0.96) | 1.66 (0.34–4.22) |
| Unweighted mean (SE^b^) | 0 | 0 | 0 | 0 | 0 | 0.59 (0.59) | 1.53 (1.52) | 2.59 (2.53) | 4.52 (3.19) | 8.90 (4.11) |
| Population-weighted mean (SE)^c^ | 0 | 0 | 0 | 0 | 0 | 2.71 (1.35) | 7.02 (3.49) | 11.68 (5.78) | 15.08 (7.28) | 19.87 (9.15) |
| **High-income countries/regions** | | | | | | | | | | |
| Number of countries | 0 | 0 | 0 | 0 | 0 | 1 | 2 | 19 | 33 | 35 |
| Median (IQR) | 0 | 0 | 0 | 0 | 0 | < 0.01 | < 0.01 | 0.00 (0.00–0.02) | 0.51 (0.13–1.40) | 2.37 (0.80–7.52) |
| Unweighted mean (SE) | 0 | 0 | 0 | 0 | 0 | 0.77 (0.77) | 2.01 (2.00) | 3.40 (3.32) | 5.92 (4.18) | 11.57 (5.34) |
| Population-weighted mean (SE) | 0 | 0 | 0 | 0 | 0 | 9.86 (2.40) | 25.58 (6.22) | 42.41 (10.31) | 54.41 (12.84) | 71.06 (15.87) |
| **Upper-middle-income countries/regions** | | | | | | | | | | |
| Number of countries | 0 | 0 | 0 | 0 | 0 | 0 | 0 | 0 | 4 | 8 |
| Median (IQR) | 0 | 0 | 0 | 0 | 0 | 0 | 0 | 0 | 0.00 (0.00–0.23) | 0.35 (0.04–0.79) |
| Unweighted mean (SE) | 0 | 0 | 0 | 0 | 0 | 0 | 0 | 0 | 0.09 (0.04) | 0.49 (0.19) |
| Population-weighted mean (SE) | 0 | 0 | 0 | 0 | 0 | 0 | 0 | 0 | 0.02 (0.03) | 0.14 (0.14) |
| **Lower-middle-income countries/regions** | | | | | | | | | | |
| Number of countries | 0 | 0 | 0 | 0 | 0 | 0 | 0 | 0 | 3 | 3 |
| Median (IQR) | 0 | 0 | 0 | 0 | 0 | 0 | 0 | 0 | 0.04 (0.02–0.05) | 0.19 (0.14–0.19) |
| Unweighted mean (SE) | 0 | 0 | 0 | 0 | 0 | 0 | 0 | 0 | 0.03 (0.02) | 0.16 (0.04) |
| Population-weighted mean (SE) | 0 | 0 | 0 | 0 | 0 | 0 | 0 | 0 | 0.04 (0.02) | 0.16 (0.04) |
| ^a^ IQR: Interquartile range;  ^b^ SE: Standard error;  ^c^ The population-weighted mean was calculated by assigning weights proportional to each country's population size for the respective year. The standard error of the weighted mean was derived from the unbiased weighted sample variance to account for the weights of different population sizes. | | | | | | | | | | |

Table S5. Consumption of preventive migraine-specific medications by income-level group, 2015-2024

**a) N02CA ergot alkaloids (lisuride)**

| Metrics | 2015 | 2016 | 2017 | 2018 | 2019 | 2020 | 2021 | 2022 | 2023 | 2024 |
| --- | --- | --- | --- | --- | --- | --- | --- | --- | --- | --- |
| **All regions** | | | | | | | | | | |
| Number of countries | 5 | 4 | 2 | 2 | 2 | 2 | 1 | 0 | 0 | 1 |
| Median (IQR^a^) | 10.90 (2.61–13.95) | 2.93 (0.01–9.68) | 0.00 (0.00–6.38) | 0.00 (0.00–4.20) | < 0.01 | < 0.01 | < 0.01 | 0 | 0 | < 0.01 |
| Unweighted mean (SE^b^) | 8.41 (2.99) | 5.29 (2.77) | 3.45 (2.23) | 1.92 (1.19) | 0.09 (0.09) | 0.05 (0.05) | < 0.01 | 0 | 0 | < 0.01 |
| Population-weighted mean (SE)^c^ | 9.27 (3.49) | 7.79 (3.45) | 5.93 (2.71) | 3.09 (1.37) | 0.06 (0.08) | 0.03 (0.04) | < 0.01 | 0 | 0 | < 0.01 |
| **High-income countries/regions** | | | | | | | | | | |
| Number of countries | 3 | 3 | 1 | 1 | 1 | 1 | 1 | 0 | 0 | 1 |
| Median (IQR) | 10.90 (6.75–12.43) | 2.93 (1.47–6.30) | 0.00 (0.00–3.19) | 0.00 (0.00–2.10) | 0.00 (0.00–0.21) | 0.00 (0.00–0.11) | < 0.01 | 0 | 0 | < 0.01 |
| Unweighted mean (SE) | 9.15 (3.39) | 4.21 (2.86) | 2.13 (2.13) | 1.40 (1.40) | 0.14 (0.14) | 0.08 (0.08) | 0.01 (0.01) | 0 | 0 | < 0.01 |
| Population-weighted mean (SE) | 7.80 (4.46) | 4.30 (3.78) | 2.76 (2.52) | 1.82 (1.66) | 0.18 (0.17) | 0.10 (0.09) | 0.01 (0.01) | 0 | 0 | < 0.01 |
| **Upper-middle-income countries/regions** | | | | | | | | | | |
| Number of countries | 0 | 0 | 0 | 0 | 0 | 0 | 0 | 0 | 0 | 0 |
| Median (IQR) | 0 | 0 | 0 | 0 | 0 | 0 | 0 | 0 | 0 | 0 |
| Unweighted mean (SE) | 0 | 0 | 0 | 0 | 0 | 0 | 0 | 0 | 0 | 0 |
| Population-weighted mean (SE) | 0 | 0 | 0 | 0 | 0 | 0 | 0 | 0 | 0 | 0 |
| **Lower-middle-income countries/regions** | | | | | | | | | | |
| Number of countries | 2 | 1 | 1 | 1 | 1 | 1 | 0 | 0 | 0 | 0 |
| Median (IQR) | 7.28 (3.64–10.92) | 6.91 (3.46–10.37) | 5.43 (2.72–8.15) | 2.70 (1.35–4.06) | < 0.01 | < 0.01 | 0 | 0 | 0 | 0 |
| Unweighted mean (SE) | 7.28 (7.28) | 6.91 (6.91) | 5.43 (5.43) | 2.70 (2.70) | < 0.01 | < 0.01 | 0 | 0 | 0 | 0 |
| Population-weighted mean (SE) | 9.98 (7.28) | 9.45 (6.91) | 7.42 (5.43) | 3.69 (2.70) | < 0.01 | < 0.01 | 0 | 0 | 0 | 0 |
| ^a^ IQR: Interquartile range;  ^b^ SE: Standard error;  ^c^ The population-weighted mean was calculated by assigning weights proportional to each country's population size for the respective year. The standard error of the weighted mean was derived from the unbiased weighted sample variance to account for the weights of different population sizes. | | | | | | | | | | |

**b) N02CD calcitonin gene-related peptide (CGRP) antagonists (atogepant, eptinezumab, erenumab, fremanezumab and galcanezumab)**

| Metrics | 2015 | 2016 | 2017 | 2018 | 2019 | 2020 | 2021 | 2022 | 2023 | 2024 |
| --- | --- | --- | --- | --- | --- | --- | --- | --- | --- | --- |
| **All regions** | | | | | | | | | | |
| Number of countries | 0 | 0 | 0 | 16 | 34 | 46 | 55 | 57 | 57 | 57 |
| Median (IQR^a^) | 0 | 0 | 0 | < 0.01 | 0.00 (0.00–0.16) | 0.04 (0.00–0.88) | 0.15 (0.03–2.61) | 0.37 (0.05–3.92) | 0.56 (0.07–5.82) | 1.30 (0.09–8.99) |
| Unweighted mean (SE^b^) | 0 | 0 | 0 | 0.06 (0.04) | 0.54 (0.20) | 1.15 (0.35) | 1.83 (0.47) | 3.39 (1.21) | 5.20 (2.12) | 9.78 (3.50) |
| Population-weighted mean (SE)^c^ | 0 | 0 | 0 | 0.15 (0.08) | 0.64 (0.32) | 0.92 (0.44) | 1.29 (0.59) | 4.49 (2.26) | 8.07 (4.11) | 12.42 (6.15) |
| **High-income countries/regions** | | | | | | | | | | |
| Number of countries | 0 | 0 | 0 | 16 | 29 | 36 | 40 | 40 | 40 | 40 |
| Median (IQR) | 0 | 0 | 0 | 0.00 (0.00–0.02) | 0.05 (0.00–0.78) | 0.18 (0.02–2.09) | 0.78 (0.11–3.20) | 1.95 (0.34–5.34) | 2.71 (0.54–6.96) | 4.61 (0.97–12.18) |
| Unweighted mean (SE) | 0 | 0 | 0 | 0.09 (0.06) | 0.78 (0.28) | 1.66 (0.48) | 2.64 (0.64) | 4.89 (1.70) | 7.52 (3.01) | 14.16 (4.94) |
| Population-weighted mean (SE) | 0 | 0 | 0 | 0.62 (0.17) | 2.58 (0.67) | 3.71 (0.89) | 5.24 (1.16) | 18.28 (4.66) | 32.85 (8.49) | 50.51 (12.56) |
| **Upper-middle-income countries/regions** | | | | | | | | | | |
| Number of countries | 0 | 0 | 0 | 0 | 3 | 6 | 10 | 12 | 12 | 12 |
| Median (IQR) | 0 | 0 | 0 | 0 | < 0.01 | < 0.01 | 0.01 (0.00–0.03) | 0.04 (0.00–0.05) | 0.03 (0.01–0.08) | 0.03 (0.01–0.11) |
| Unweighted mean (SE) | 0 | 0 | 0 | 0 | < 0.01 | 0.01 (0.01) | 0.03 (0.02) | 0.05 (0.02) | 0.06 (0.02) | 0.07 (0.02) |
| Population-weighted mean (SE) | 0 | 0 | 0 | 0 | < 0.01 | 0.01 (0.01) | 0.02 (0.02) | 0.03 (0.02) | 0.03 (0.02) | 0.04 (0.03) |
| **Lower-middle-income countries/regions** | | | | | | | | | | |
| Number of countries | 0 | 0 | 0 | 0 | 2 | 4 | 5 | 5 | 5 | 5 |
| Median (IQR) | 0 | 0 | 0 | 0 | < 0.01 | 0.00 (0.00–0.02) | 0.02 (0.01–0.03) | 0.03 (0.00–0.03) | 0.01 (0.00–0.02) | 0.01 (0.00–0.02) |
| Unweighted mean (SE) | 0 | 0 | 0 | 0 | < 0.01 | 0.02 (0.02) | 0.04 (0.03) | 0.03 (0.01) | 0.01 (0.01) | 0.01 (0.01) |
| Population-weighted mean (SE) | 0 | 0 | 0 | 0 | < 0.01 | < 0.01 | < 0.01 | < 0.01 | < 0.01 | < 0.01 |
| ^a^ IQR: Interquartile range;  ^b^ SE: Standard error;  ^c^ The population-weighted mean was calculated by assigning weights proportional to each country's population size for the respective year. The standard error of the weighted mean was derived from the unbiased weighted sample variance to account for the weights of different population sizes. | | | | | | | | | | |

**c) N02CX other antimigraine preparations (clonidine, dimetotiazine, iprazochrome, oxetorone and pizotifen)**

| Metrics | 2015 | 2016 | 2017 | 2018 | 2019 | 2020 | 2021 | 2022 | 2023 | 2024 |
| --- | --- | --- | --- | --- | --- | --- | --- | --- | --- | --- |
| **All regions** | | | | | | | | | | |
| Number of countries | 40 | 39 | 40 | 39 | 39 | 36 | 36 | 35 | 32 | 33 |
| Median (IQR^a^) | 49.79 (10.90–295.08) | 39.66 (8.27–263.74) | 47.21 (6.90–260.35) | 39.90 (5.32–245.66) | 26.18 (1.31–224.25) | 16.79 (0.05–216.41) | 17.85 (0.29–199.75) | 17.53 (0.03–228.98) | 22.81 (0.00–203.22) | 29.14 (0.00–199.38) |
| Unweighted mean (SE^b^) | 252.68 (62.67) | 258.44 (75.15) | 248.39 (75.98) | 212.18 (51.54) | 174.45 (47.40) | 162.70 (44.14) | 156.14 (43.08) | 155.58 (41.49) | 145.40 (37.60) | 145.99 (37.52) |
| Population-weighted mean (SE)^c^ | 96.31 (41.88) | 95.22 (41.88) | 89.62 (39.82) | 91.15 (37.87) | 86.04 (36.16) | 79.31 (31.25) | 73.61 (31.17) | 85.22 (31.78) | 86.19 (30.66) | 86.14 (30.69) |
| **High-income countries/regions** | | | | | | | | | | |
| Number of countries | 24 | 22 | 23 | 23 | 23 | 21 | 21 | 20 | 18 | 21 |
| Median (IQR) | 100.42 (25.55–407.87) | 79.01 (17.29–391.04) | 88.21 (16.96–374.97) | 86.16 (16.21–379.39) | 40.75 (12.88–268.26) | 38.18 (5.24–236.27) | 26.88 (5.00–262.52) | 37.91 (0.05–256.56) | 35.42 (0.00–243.18) | 66.36 (2.97–251.83) |
| Unweighted mean (SE) | 346.16 (97.07) | 358.50 (121.22) | 345.66 (123.72) | 267.20 (75.67) | 215.04 (71.45) | 190.04 (62.03) | 192.67 (63.76) | 187.23 (61.62) | 169.59 (53.90) | 176.39 (55.37) |
| Population-weighted mean (SE) | 244.87 (93.30) | 239.64 (93.89) | 225.45 (89.58) | 213.02 (80.04) | 205.50 (79.10) | 182.20 (65.12) | 192.87 (67.40) | 196.80 (66.67) | 194.01 (62.15) | 200.54 (63.29) |
| **Upper-middle-income countries/regions** | | | | | | | | | | |
| Number of countries | 9 | 10 | 10 | 9 | 9 | 8 | 8 | 8 | 7 | 7 |
| Median (IQR) | 11.69 (0.05–42.58) | 6.76 (0.04–34.75) | 3.20 (0.02–41.64) | 2.23 (0.01–76.18) | 1.14 (0.00–39.10) | 1.05 (0.00–30.64) | 1.17 (0.00–39.73) | 0.39 (0.00–29.91) | 0.44 (0.00–28.13) | 0.96 (0.00–22.47) |
| Unweighted mean (SE) | 71.65 (44.49) | 72.90 (47.33) | 74.39 (48.71) | 112.87 (79.58) | 78.15 (51.32) | 73.65 (48.13) | 70.28 (44.62) | 70.08 (46.22) | 68.48 (46.94) | 68.60 (47.85) |
| Population-weighted mean (SE) | 15.29 (25.89) | 15.40 (27.39) | 15.68 (28.27) | 23.53 (45.33) | 16.89 (30.18) | 16.15 (28.60) | 15.89 (26.78) | 15.84 (27.71) | 15.54 (27.79) | 16.19 (28.26) |
| **Lower-middle-income countries/regions** | | | | | | | | | | |
| Number of countries | 7 | 7 | 7 | 7 | 7 | 7 | 7 | 7 | 7 | 5 |
| Median (IQR) | 74.39 (4.96–195.81) | 80.35 (5.48–207.51) | 77.81 (4.20–187.91) | 26.37 (0.33–203.75) | 25.55 (3.88–199.07) | 28.88 (1.53–194.50) | 26.40 (2.77–104.98) | 24.45 (1.15–213.00) | 25.55 (5.07–207.30) | 23.42 (0.00–196.46) |
| Unweighted mean (SE) | 186.85 (110.34) | 177.66 (97.80) | 161.89 (87.47) | 164.38 (103.36) | 167.98 (105.42) | 188.58 (126.64) | 149.31 (105.70) | 163.56 (98.21) | 165.96 (97.75) | 147.72 (86.04) |
| Population-weighted mean (SE) | 143.50 (56.76) | 144.73 (53.10) | 132.59 (48.32) | 134.40 (54.49) | 138.36 (56.33) | 136.41 (61.98) | 88.08 (50.07) | 149.09 (56.67) | 158.15 (60.60) | 144.41 (53.60) |
| ^a^ IQR: Interquartile range;  ^b^ SE: Standard error;  ^c^ The population-weighted mean was calculated by assigning weights proportional to each country's population size for the respective year. The standard error of the weighted mean was derived from the unbiased weighted sample variance to account for the weights of different population sizes. | | | | | | | | | | |

Figure S1a. Trends in unweighted average consumption of migraine-specific medications by income-level group, 2015-2024


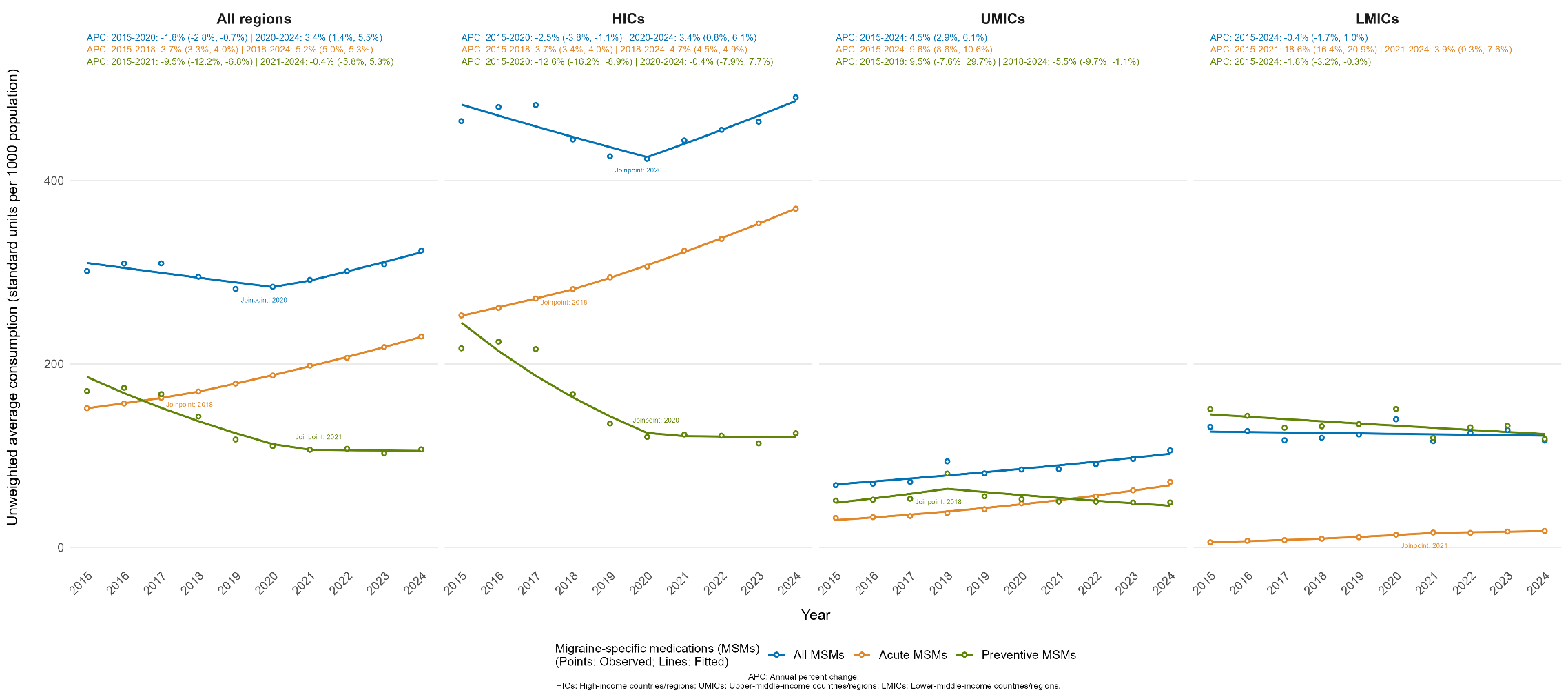


Figure S1b. Trends in median consumption of migraine-specific medications by income-level group, 2015-2024


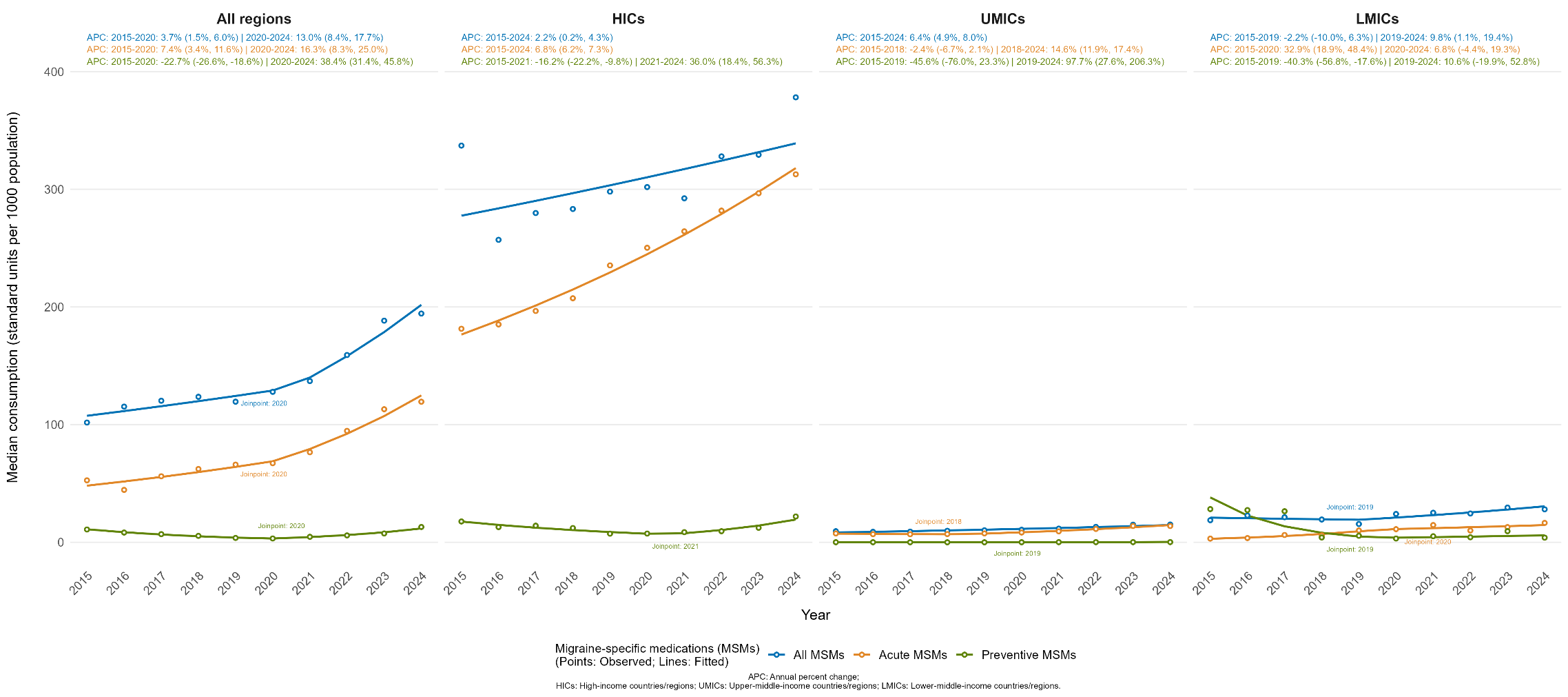


Figure S2a. Trends in unweighted average consumption of acute migraine-specific medications by income-level group, 2015-2024


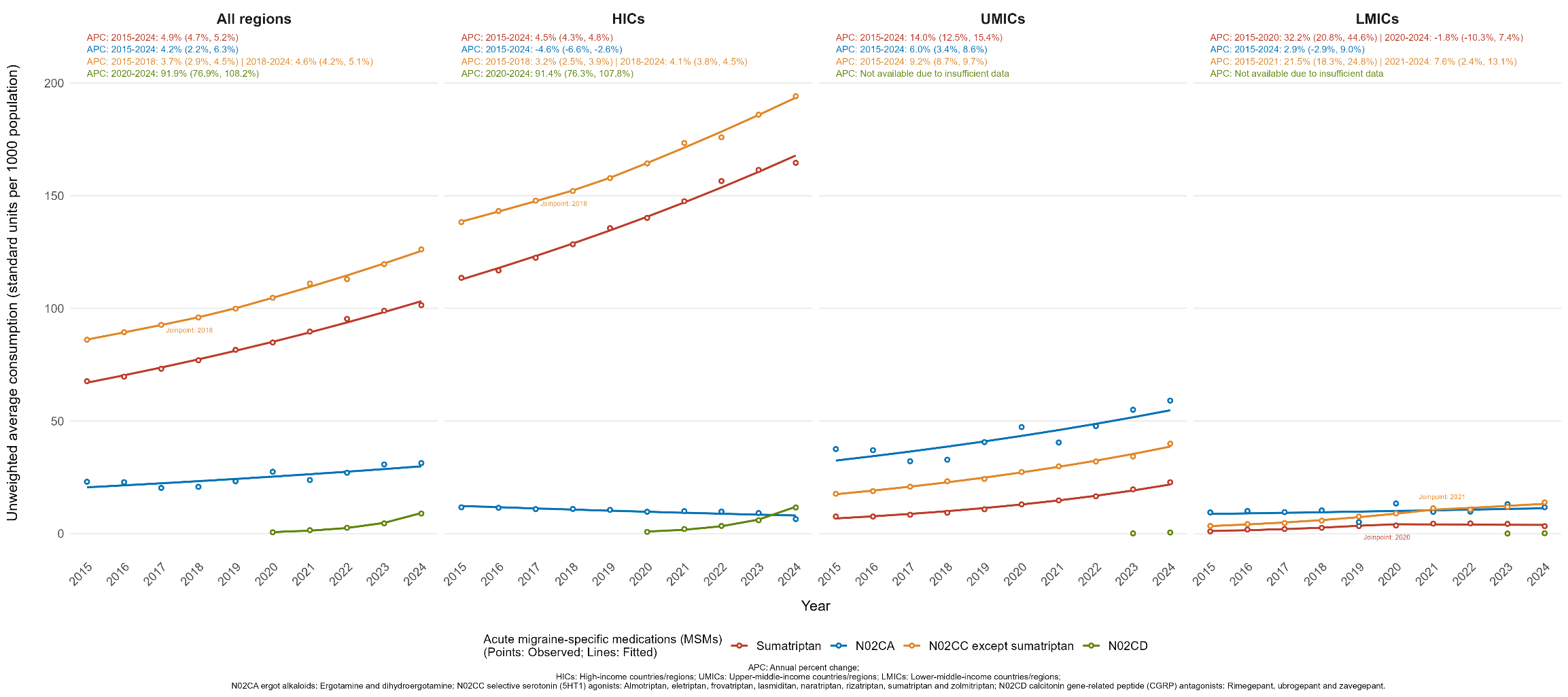


Figure S2b. Trends in median consumption of acute migraine-specific medications by income-level group, 2015-2024


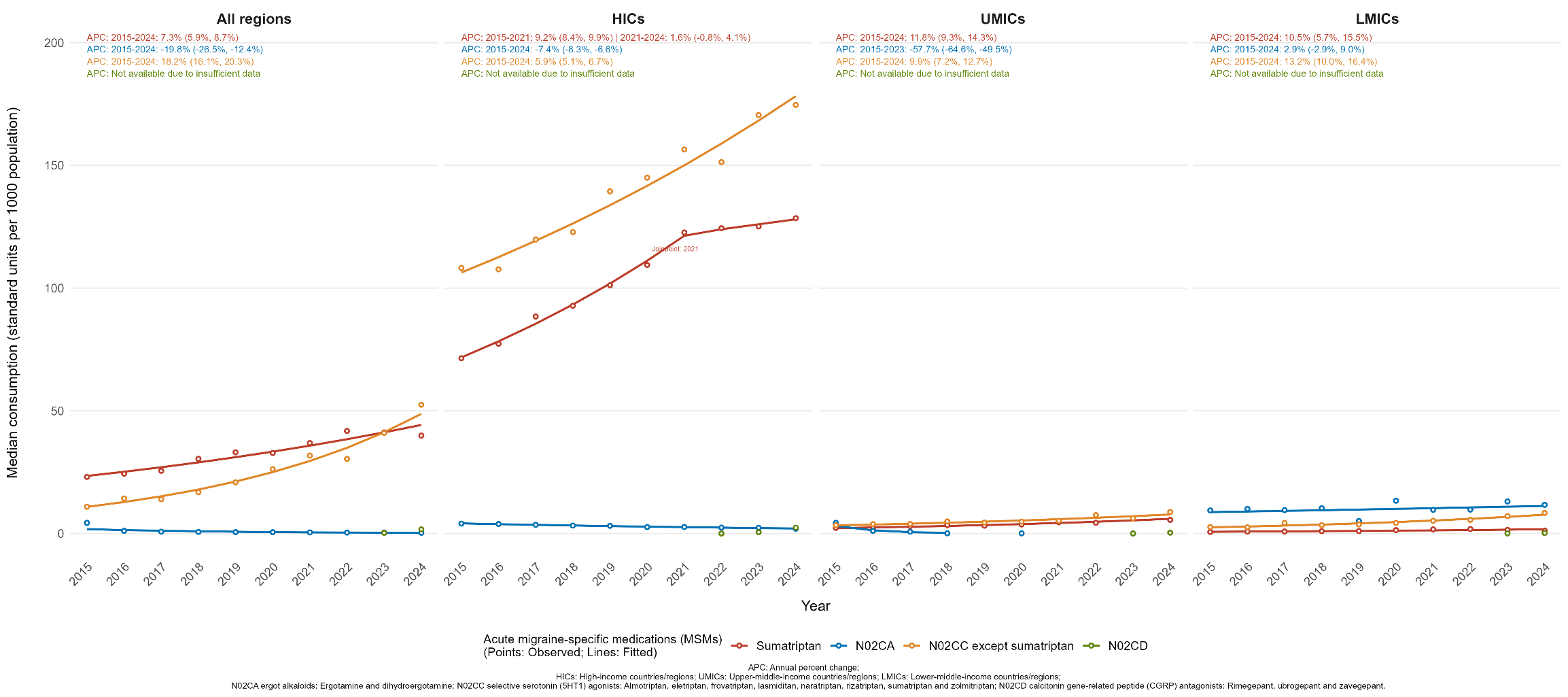


Figure S3a. Trends in unweighted average consumption of preventive migraine-specific medications by income-level group, 2015-2024


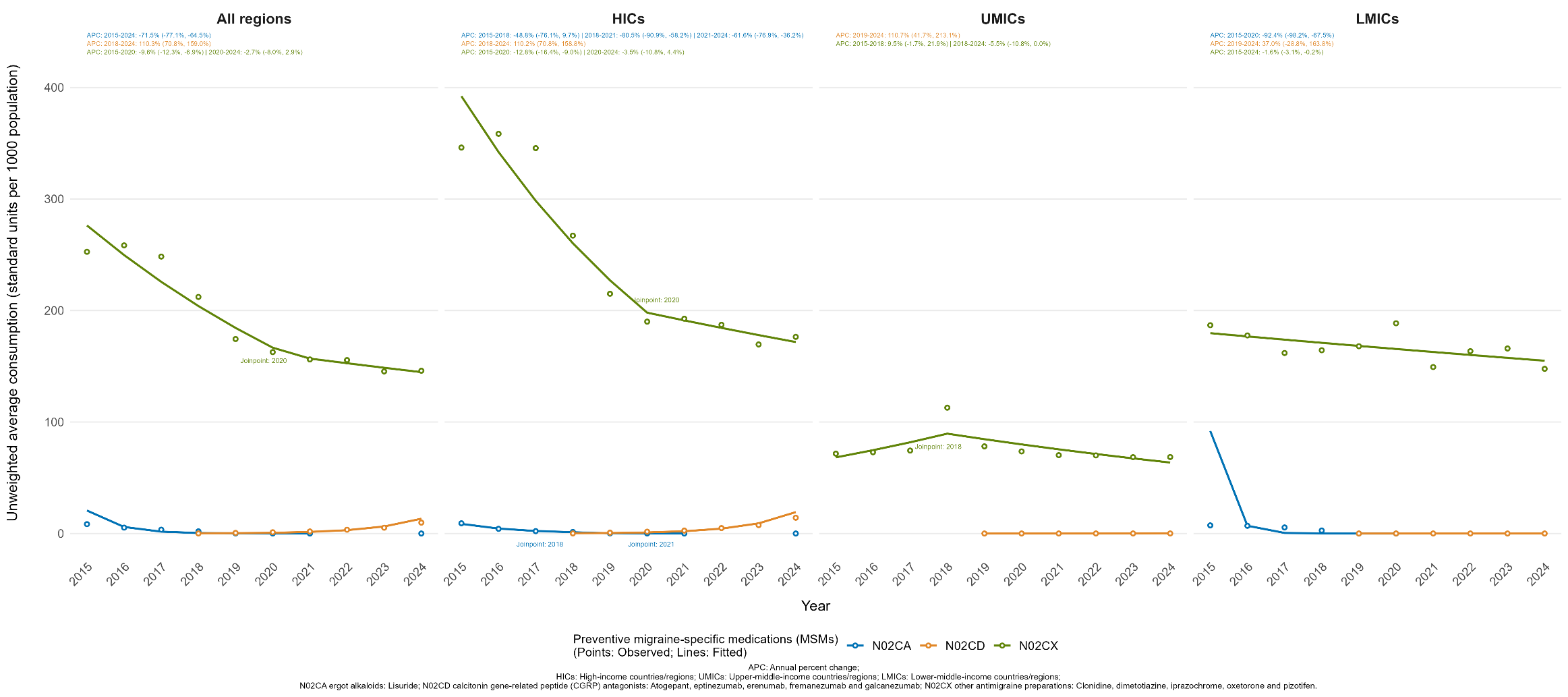


Figure S3b. Trends in median consumption of preventive migraine-specific medications by income-level group, 2015-2024


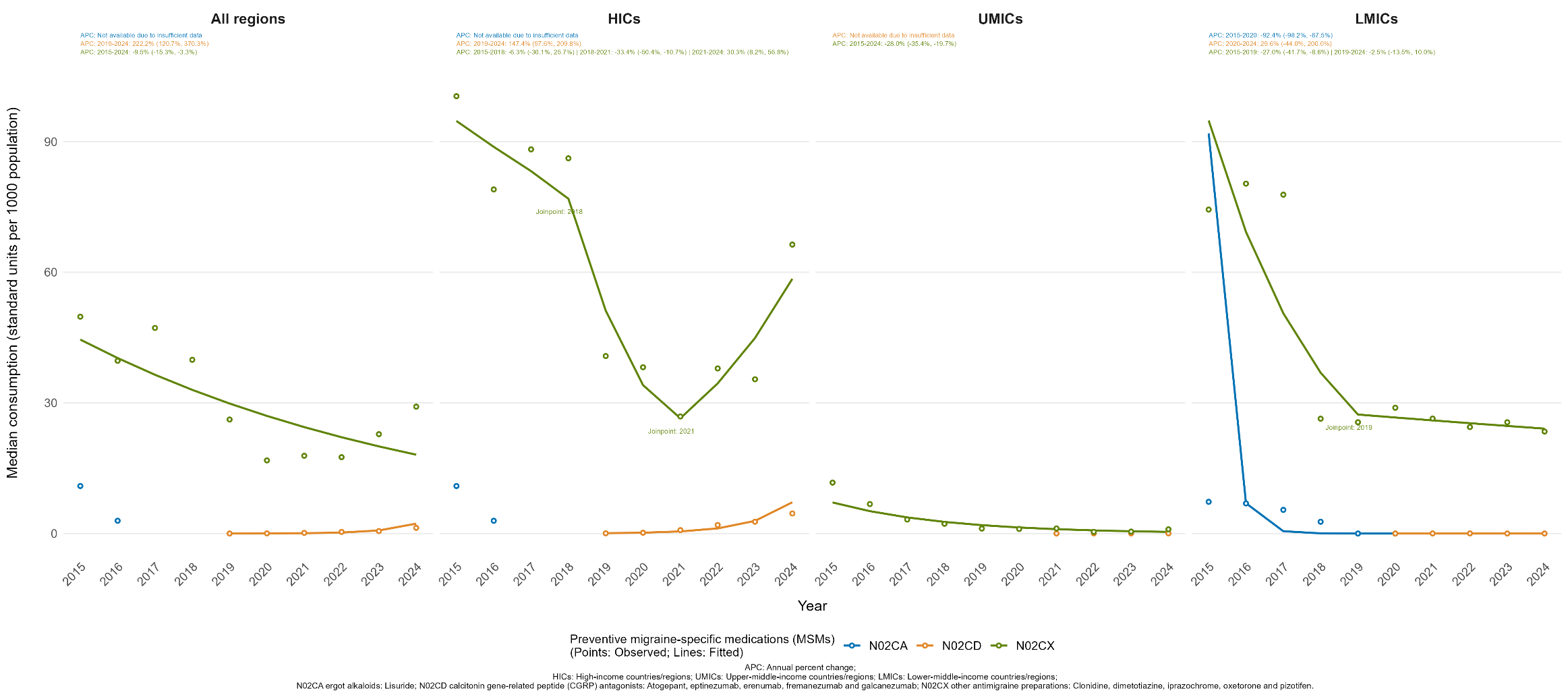


Figure S4a. Trends in population-weighted average consumption of propranolol by income-level group, 2015-2024


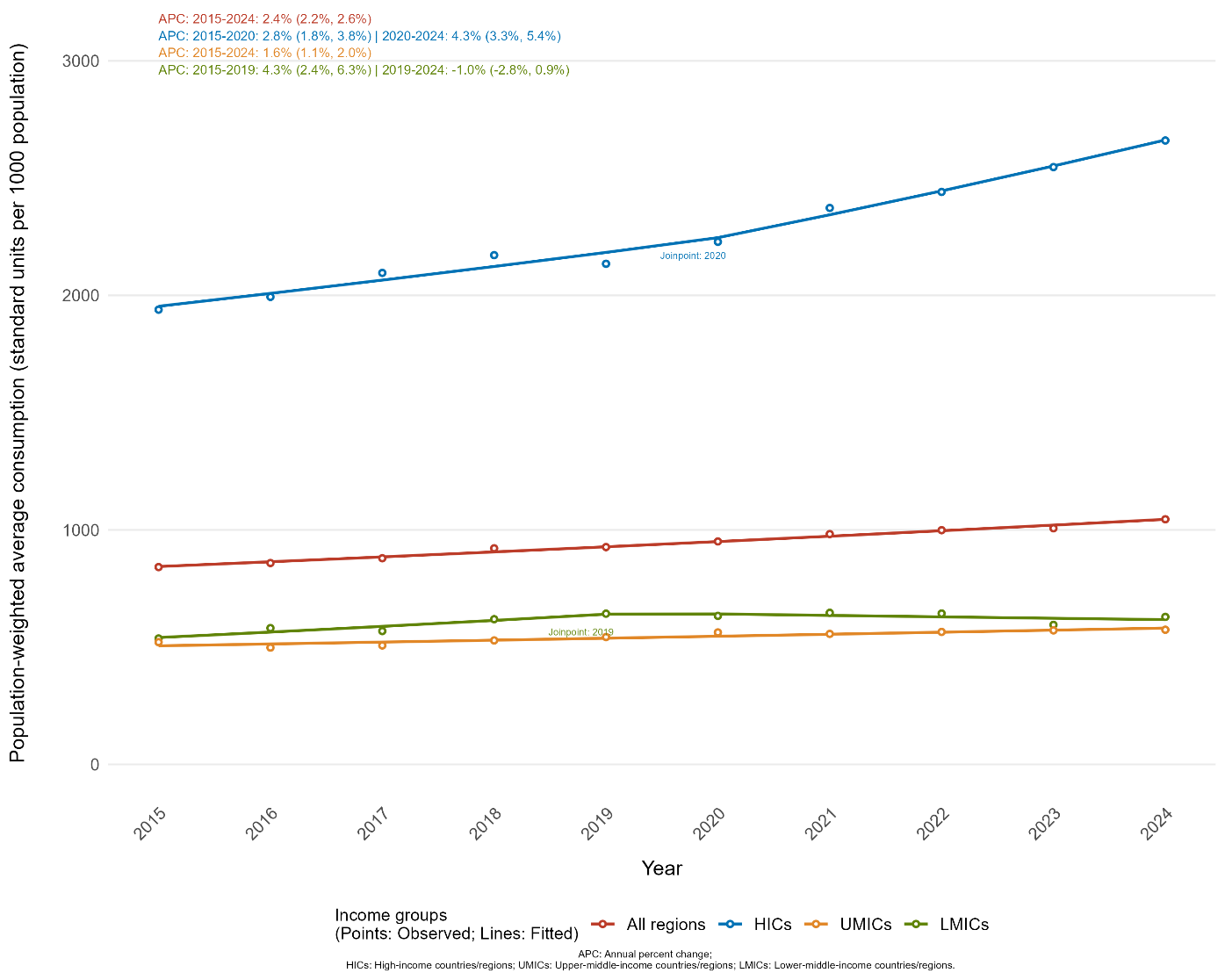


Figure S4b. Trends in unweighted average consumption of propranolol by income-level group, 2015-2024


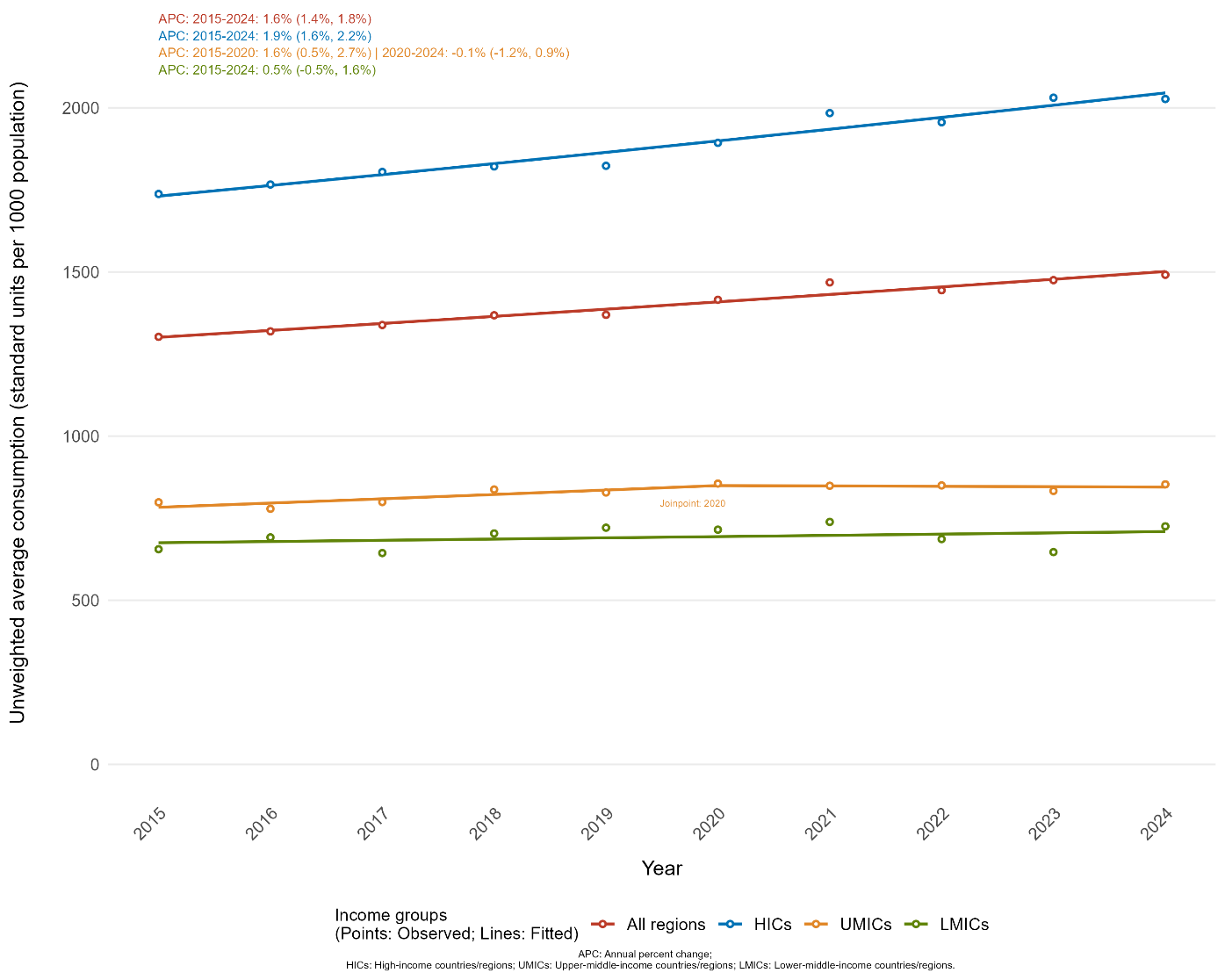


Figure S4c. Trends in median consumption of propranolol by income-level group, 2015-2024


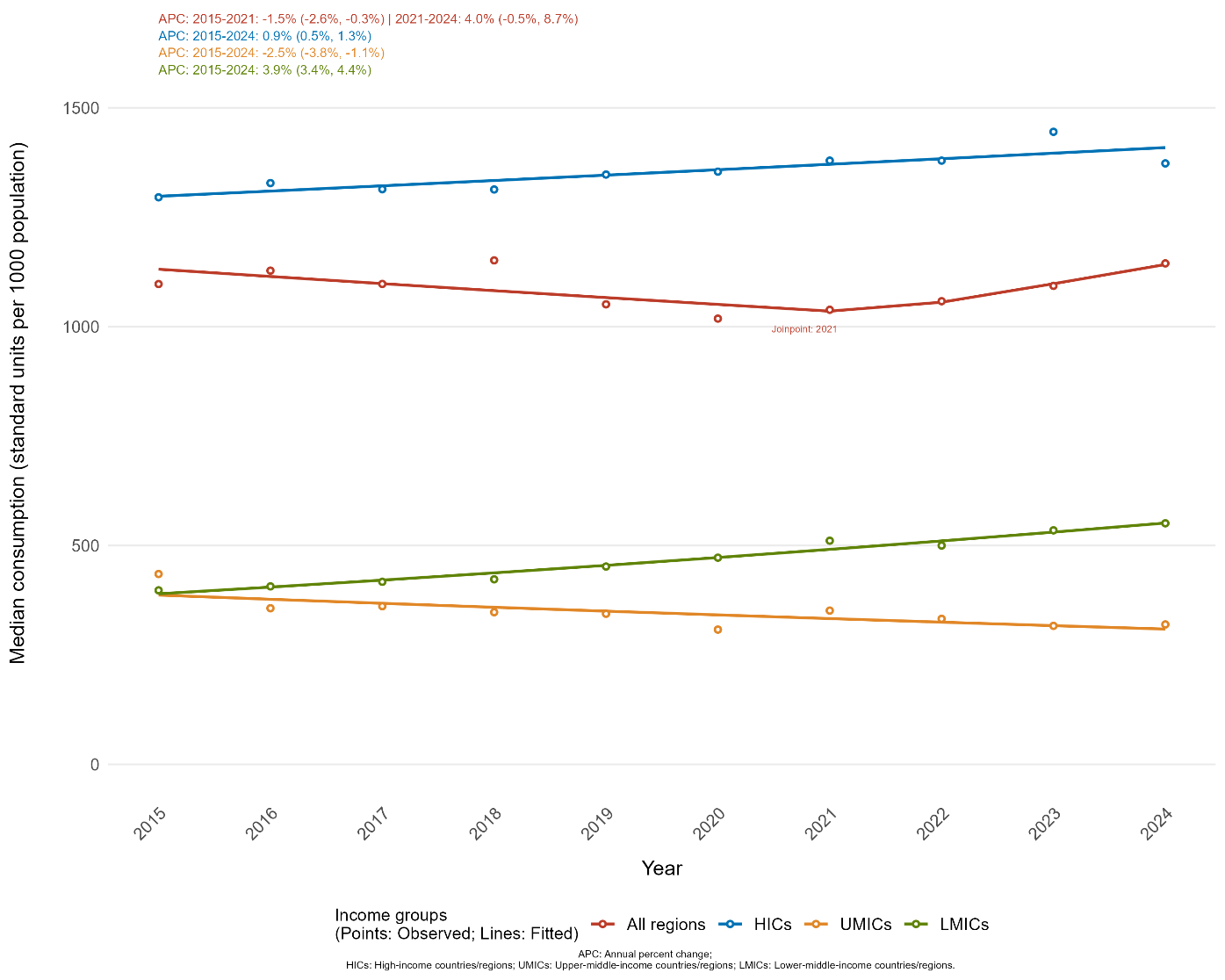


Figure S5. Impact of introduction of the first calcitonin gene-related peptide (CGRP) inhibitor and COVID-19 onset on non-CGRP migraine-specific medication consumption stratified by country, 2015-2024


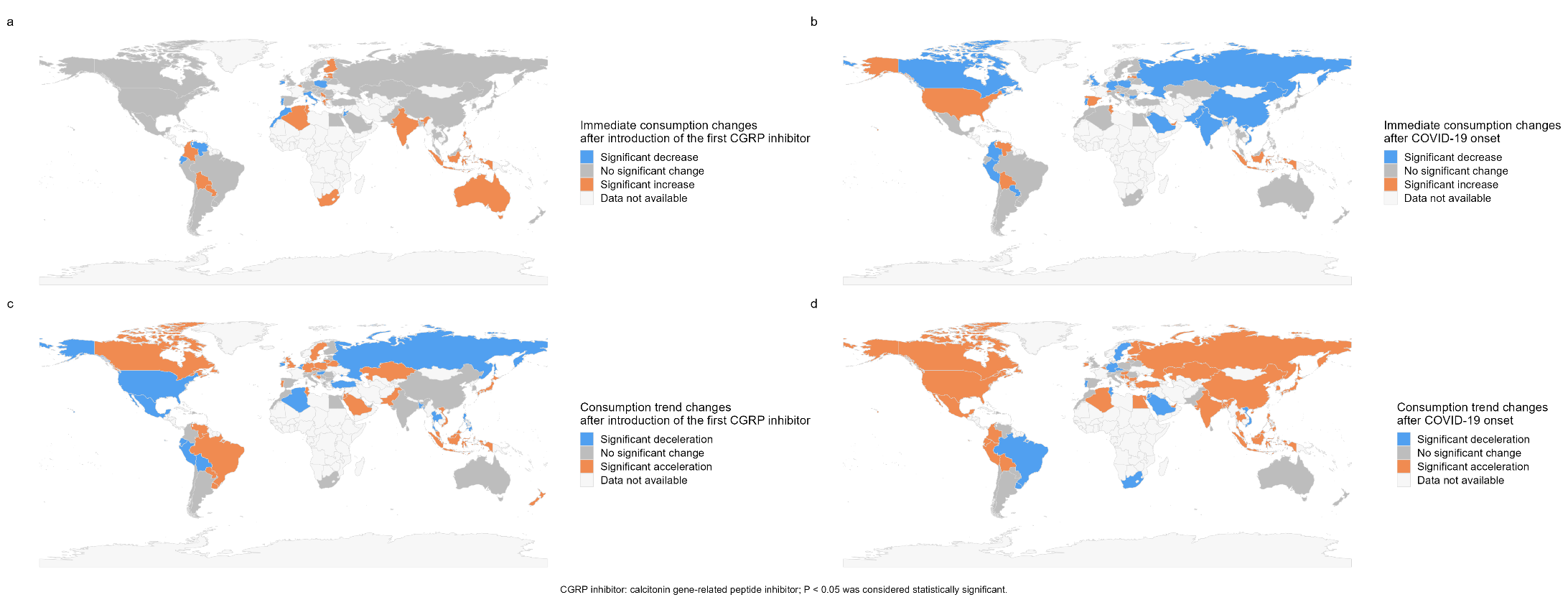


Figure S6. Impact of introduction of the first calcitonin gene-related peptide (CGRP) inhibitor and COVID-19 onset on non-CGRP acute migraine-specific medication consumption stratified by country, 2015-2024


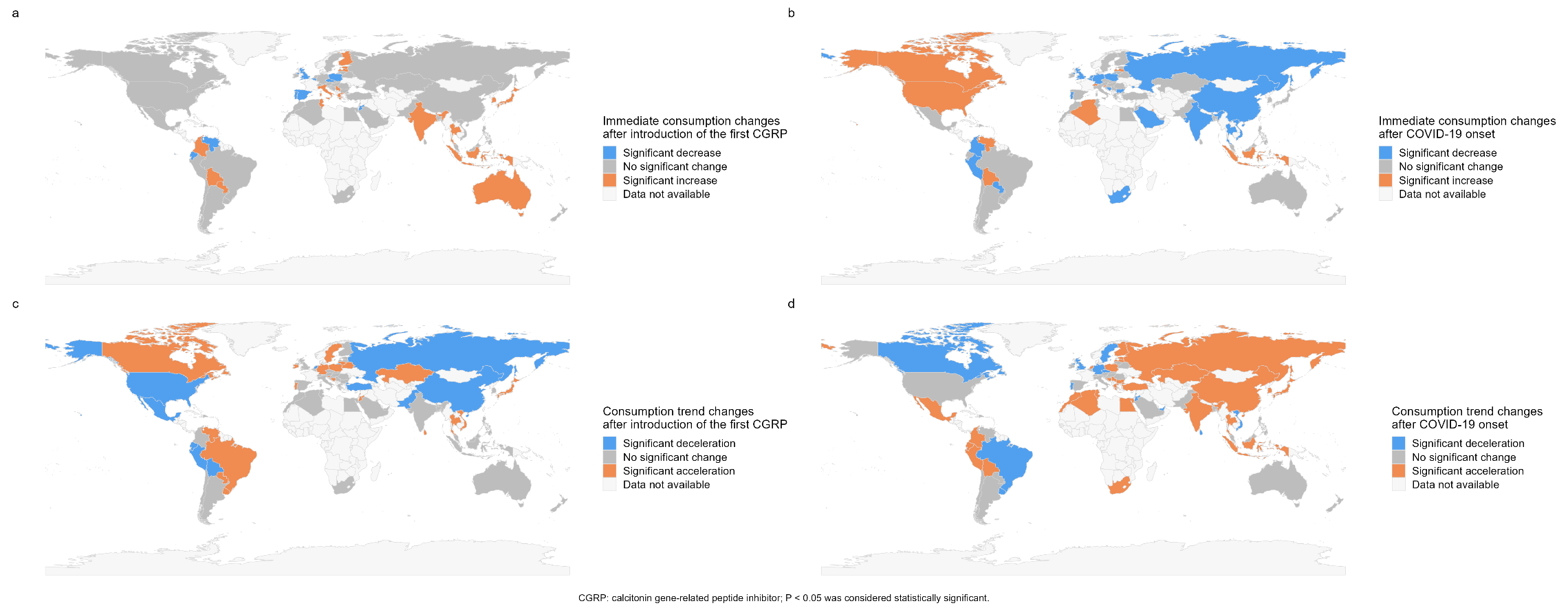


Figure S7. Impact of introduction of the first calcitonin gene-related peptide inhibitor and COVID-19 onset on propranolol consumption stratified by country, 2015-2024


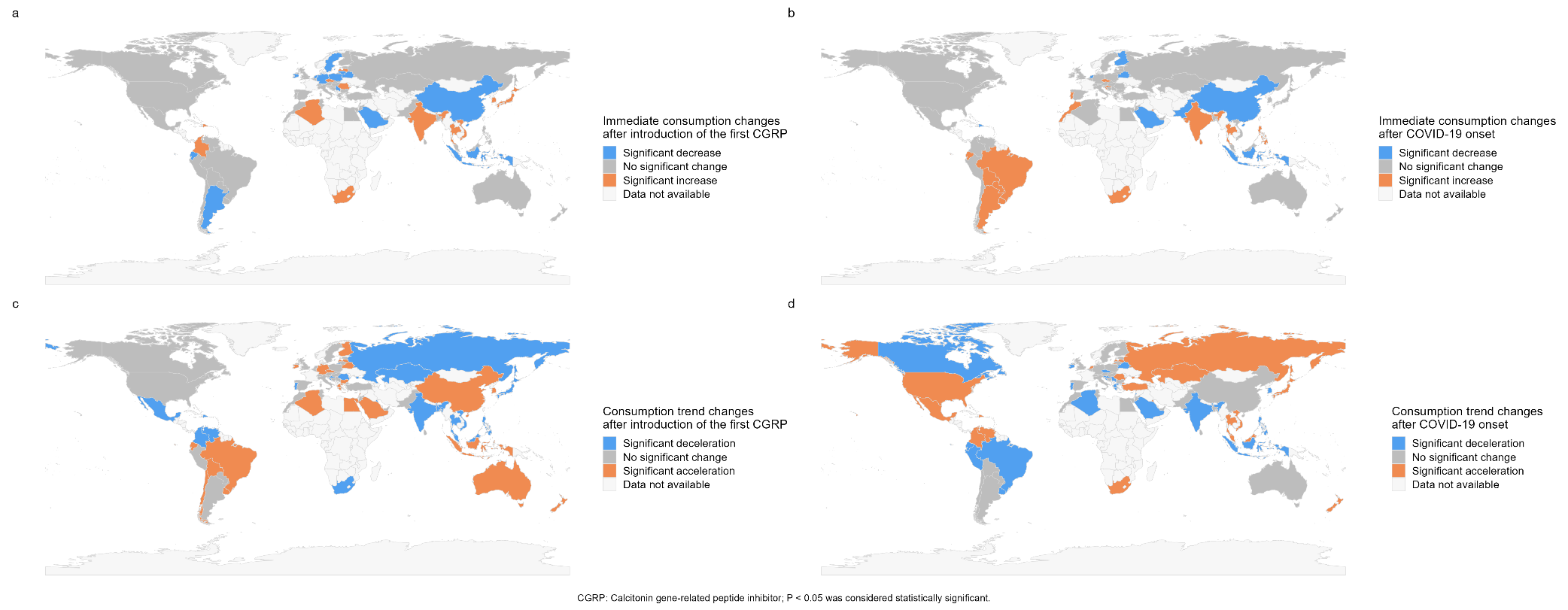

Supplement: Supplementary file 1 — Supplementary Material 1 [file 10194_2026_2363_MOESM1_ESM.docx]
